# A distributed analysis approach for pharmacovigilance data from electronic medical records in German university hospitals: the POLAR_MI ETL Pipeline

**DOI:** 10.1186/s12911-026-03550-w

**Published:** 2026-06-15

**Authors:** Miriam Kesselmeier, Torsten Thalheim, Florian Schmidt, Thomas Peschel, Julia Palm, Alexander Strübing, André Medek, Jens Przybilla, Anna Maria Wermund, Renke Maas, Steffen Härterich, Louisa Redeker, Martin Federbusch, Daniel Steinbach, Jan Gewehr, Marcus Wurlitzer, Andrea Riedel, Frank Meineke, Daniel Neumann, André Scherag, Markus Loeffler

**Affiliations:** 1 Institute of Medical Statistics, Computer and Data Sciences (IMSID), Jena University Hospital-Friedrich Schiller University Jena, Jena, Germany; 2 Interdisciplinary Centre for Bioinformatics, Leipzig University, Leipzig, Germany; 3 Institute for Medical Informatics, Statistics and Epidemiology (IMISE), Leipzig University, Leipzig, Germany; 4 Deutsches Biomasseforschungszentrum gGmbH, Torgauer Str. 116, 04347 Leipzig, Germany; 5 Medical & Scientific Technology Development & Coordination (MWTek), University Hospital Bonn, Bonn, Germany; 6 Clinical Trial Centre Leipzig (ZKS), Leipzig University, Leipzig, Germany; 7 Department of Clinical Pharmacy, Institute of Pharmacy, University of Bonn, Bonn, Germany; 8 Institute of Experimental and Clinical Pharmacology and Toxicology, Friedrich-Alexander-Universität Erlangen-Nürnberg, Erlangen, Germany; 9 Hospital Pharmacy, University Medical Center Hamburg-Eppendorf, Hamburg, Germany; 10 Department of Clinical Pharmacology, School of Medicine, Faculty of Health, Witten/Herdecke University, Witten, Germany; 11 Institute for Laboratory Medicine, Clinical Chemistry and Molecular Diagnostics, University Medical Center Leipzig, Leipzig, Germany; 12 Business Division for Information Technology, University Medical Center Hamburg-Eppendorf, Hamburg, Germany; 13 Erlangen University Hospital, Medical Center for Information and Communication Technology, Erlangen, Germany; 14 Friedrich-Alexander-Universität Erlangen-Nürnberg, Medical Informatics, Erlangen, Germany

**Keywords:** Distributed analysis, Distributed computation, Meta-analysis, Data integration, ETL, Extract transform load, Lessons learned, Hospital information system, Electronic medical records, Electronic health records

## Abstract

**Background:**

The cooperative project “POLypharmacy, drug interActions and Risks” (POLAR_MI) of the Medical Informatics Initiative Germany (MII) aimed at detecting medication-related risks attributed to polymedication in adult patients from German university hospitals. Here, we report technological challenges and solutions to undertake this large-scale multicentre project relying on routine healthcare data stored and processed by the data integration centres, which were recently established at the German university hospitals.

**Methods:**

We developed and implemented a two-step, privacy-preserving, distributed analysis approach to analyse clinical routine healthcare data relying on the internationally balloted MII HL7^®^ FHIR^®^ core data set specifications (version 1.0). In this approach, without direct data access for the data analysts, a local data aggregation step comprising data extraction, transformation (including statistical analyses) and loading (ETL) at each university hospital’s data integration centre was followed by a central random-effects meta-analysis.

**Results:**

Using an iterative procedure between data integration centres and the cross-institutional analysis team, we overcame many challenges and established the “POLAR_MI ETL Pipeline”. These challenges originated from the heterogeneity of the data integration centres and the IT infrastructure of the related university hospitals including their local hospital information system. Applying our pipeline, we analysed data from ten centres on nearly 800,000 encounters from about 500,000 patients.

**Conclusions:**

For the first time within the MII infrastructure, we demonstrated that a project on routine healthcare data is feasible using a distributed analysis approach based on the recently established network of data integration centres in Germany. We describe an approach to obtain a valuable and insightful overview of health risks in routine healthcare and share the related code. Moreover, we propose improvements to the ETL process for future distributed analyses. Finally, our data-related challenges and solutions can be adapted to other healthcare settings (in other countries and initiatives, respectively) as long as data integration centres equivalents and a common data format/model are available.

**Trial registration:**

POLAR_MI was registered on 27/11/2020 in the “HMA-EMA Catalogues of real-world data sources and studies” (EU PAS number: EUPAS36582).

**Supplementary Information:**

The online version contains supplementary material available at 10.1186/s12911-026-03550-w.

## Background

In 2021, Germany had 36 university hospitals supporting a population of about 83 million citizens [1]. These hospitals are independent public institutions to serve the general population’s medical needs. They care for about two million inpatients (about 11% of all hospitalisations in Germany) and for an additional twelve million outpatients each year. Each university hospital runs its independent hospital information system (HIS) and, so far, there has been little harmonisation and data exchange between them. The Medical Informatics Initiative Germany (MII [2]) aims at building up an infrastructure to support healthcare research with patient data from hospital electronic medical records (EMR) of all German university hospitals. The aim is to constitute a network with a unified data extraction and usage framework. For this purpose, data integration centres (DIC) were established at each university hospital [3]. They rely on coordinated, common technical and organisational prerequisites. Examples are the agreement to use HL7^®^ FHIR^®^ (Health Level Seven Fast Health Interoperability Resources [4]) as an interoperable data format, which is further qualified by the profiles defined in the so-called MII core data set (CDS) specifications [5, 6]. These profiles are a composition of different, interdependent resources, e.g., general information on the patient/encounter, medications, conditions (including diagnoses and procedures) and laboratory values.

Within the MII, the cooperative use case “POLypharmacy, drug interActions and Risks” (POLAR_MI [7]) was designed to detect medication-related risks in adult inpatients (aged ≥ 18 years) with polymedication. Especially the hospitalised population is often confronted with the required intake of more than one medication, which increases the risk for medication-related problems, including potentially inadequate medications, contraindications and adverse drug events (ADE) [8]. In POLAR_MI, we addressed five research projects, which were related to health risks imposed by (i) potentially inappropriate medication, (ii) contraindicated drug prescriptions, (iii) potentially inadequate prescribing in patients with renal insufficiency as well as (iv) emergency hospital admissions and re-admissions related to suspected ADE and (v) to the development of models predicting ADE. To reach these aims, researchers from different disciplines (e.g., computer sciences, medical biometry, epidemiology, (clinical) pharmacology, pharmacy and health science) worked together to develop and establish a privacy-preserving distributed analysis approach (hereafter abbreviated as “POLAR_MI ETL Pipeline”, where ETL stands for **e**xtract, **t**ransform and **l**oad data).

In this article, we focus on the challenge of implementing the POLAR_MI ETL Pipeline at German university hospitals. After describing the general layout of this pipeline, we focus on generalisable lessons learned and our handling of these challenges, particularly with respect to the method to retrieve and process EMR data locally at the DIC without direct data access by the analysts. These lessons learned also include considerations on how to formulate research questions that can be answered with data from routine healthcare and on required assumptions to answer such research questions. Finally, we provide selected results from a real data application within POLAR_MI for illustrative purposes and to quantify the frequency of several challenges. Complete results from the above-mentioned POLAR_MI research projects are and will be reported independently [9, 10].

## Methods

### Distributed analysis approach

**Fig. 1:**
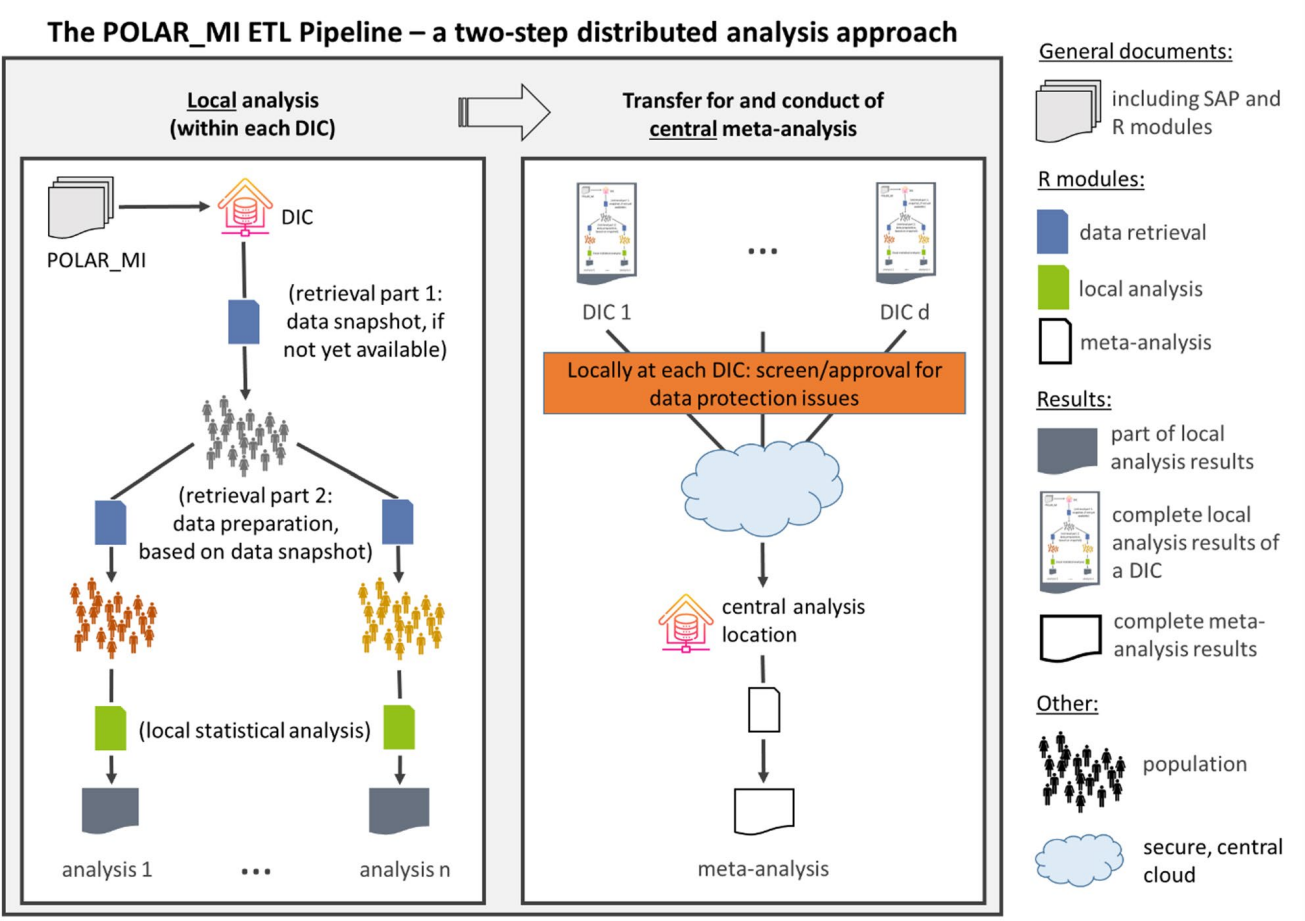
The POLAR_MI ETL Pipeline. The distributed analysis approach in POLAR_MI consists of a local analysis (left panel) with subsequent central meta-analysis (right panel). The R modules to be executed locally were made available to the DIC via an internal GitLab repository. The data snapshot within retrieval part 1 is equivalent to data extraction, but is only created once prior to the first analysis and used for all subsequent analyses. The data preparation (retrieval part 2) and the local statistical analysis start at the available data snapshot and are conducted sequentially. For details on the queries via the R package *fhircrackr* [22] within retrieval part 1, we refer to Supplementary Fig. 1 (Additional File 3). Abbreviations: d, number of different DIC; DIC, data integration centre; ETL, extract, transform, load; FHIR, Fast Health Interoperability Resources; n, number of different analyses; SAP, statistical analysis plan

The distributed analysis approach was developed to analyse routine healthcare data retrospectively. Within the selected approach, patient consent was not required according to German law. The approach (Fig. 1) consisted of several steps. Starting from the CDS-compliant FHIR data (during POLAR_MI: CDS version 1.0 [11–15]), centrally provided data retrieval and analysis software modules were applied within each DIC. The retrieval module downloaded the data from the local FHIR server and performed basic data pre-processing; the analysis module then aggregated the data locally. Both modules were an assembly of R scripts, prepared centrally by the multidisciplinary, cross-institutional POLAR_MI analysis team and provided to the DIC via an internal GitLab™ repository [16]. For the local execution of the data retrieval and analysis software modules, the DIC had the choice of either running a centrally provided and locally configured Docker^®^ container containing its own R environment and the modules [17–19] or of manually executing the modules on a computer with access to the FHIR server. Secondly, after running the two modules locally at each DIC, the aggregated data was screened for data protection issues locally and, when approved, uploaded to a secure, central cloud with clearly defined access regulations. Finally, a meta-analysis was performed centrally. An overview of the R packages, which have been used in the locally executed step of this approach, is provided in Supplementary Table 1 (Additional File 2) [20–41]. The R modules developed for analyses within POLAR_MI accompany the respective publications [9, 10] and are made available via the “Health Atlas - Local Data Hub/Leipzig” (see: availability of data and materials).

### Local statistical analysis at each data integration centre

First, a data snapshot was taken at each DIC comprising patients and information of their hospital stays. This data snapshot was a copy of all data available on the DIC’s local FHIR server related to inpatient hospital stays within the POLAR_MI analysis period. For the technical realisation of the data snapshot at each DIC, a retrieval request procedure was implemented (Supplementary Fig. 1, Additional File 3). The requests were sent to the local FHIR server to select and download the required pseudonymised resources. Then, this data was transformed from the FHIR format to a table format using the R package *fhircrackr* [22]. Within this process, one table with predefined items was created for each relevant resource and saved separately. This had to be done only once in conjunction with the first analysis. In the FHIR data, each hospital stay of a patient was integrated as one or, induced by the in-house workflow, as multiple so-called “encounter(s)”.

Given the data snapshot, data was prepared for the statistical analysis. The additional data preparation comprised the following tasks:

- to select items/variables and calculate/deduce additional pre-specified variables (e.g., age, specific disease, categorisation of laboratory values, specific medication)
- to apply the general POLAR_MI and, if required, additional analysis-specific inclusion and exclusion criteria
- to check for technical exclusions (Supplementary Table 2, Additional File 2; Supplementary Fig. 2, Additional File 3)
- to perform automatic plausibility checks (see below)
- to create the analysis-specific tables building the starting point of the succeeding analysis module

The tables generated in this step contained pseudonymised data. Based on these tables, the local statistical analysis (including further plausibility checks) was conducted to create aggregated data without encounter- and patient-identifying data. The statistical analysis was not limited to descriptive statistics (e.g., counts, frequencies, median) but also included association analyses (e.g., logistic regression modelling including model evaluation). As examples for the required preparations and harmonisations, we refer to the POLAR_MI-associated works [9, 10].

To identify (and subsequently resolve) incidents during analysis, we implemented plausibility checks in all modules. Critical incidents halted the entire analysis (example: contradictions induced by two or more variables). In case of minor incidents, the analysis could continue with a warning (example: value out of expected range). To collect this and other information (e.g., module run-time, analysis progress and errors/warnings), we have implemented a logging framework that provides detailed, meaningful information about problems that occurred during the execution of the modules.

### Central meta-analysis of local results

Local results were checked for plausibility (e.g., number of encounters relative to the time frame included in the analysis, frequency of events in comparison to literature, frequency and pattern of missing data, frequency of numerical issues in case of regression modelling). Local re-runs were requested in case of missing, incomplete or (suspected) incorrect results. Subsequently, it was checked whether local results could be included in the meta-analysis. For example, in case of logistic regression modelling, the inclusion of local results was only appropriate if the regression model estimation converged properly (suggesting numerically stable results). Once the centres to be included were identified, a random-effects meta-analysis (to account for hospital and DIC heterogeneity) was performed. An application of this approach with all required details is provided in the POLAR_MI-associated articles [9, 10].

### Application to POLAR_MI data

The POLAR_MI’s overall workflow and development cycle for the operationalisation of each POLAR_MI research project is presented in Supplementary Fig. 3 (Additional File 3). All POLAR_MI research projects relied on the general “POLAR_MI population”, which was characterised by the following inclusion criteria:

- Admission and discharge within the time interval 2018/01/01 and 2021/12/31 (in the strict sense: both encounter start and encounter end date within the time interval)
- Inpatient hospital stay (Supplementary Table 3, Additional File 2)
- Patient age of ≥ 18 years at admission and born after 1910/01/01 (Supplementary Table 2, Additional File 2)

Besides these inclusion criteria, there were additional exclusion criteria related to technical issues hindering data processing (Supplementary Table 2, Additional File 2) [42, 43]. The exclusion of the affected encounters from the POLAR_MI population resulted in the modified POLAR_MI population. In addition, each research project had additional exclusion criteria, which are provided in the respective publications [9, 10]. An overview of populations, which were considered within POLAR_MI, is provided in Supplementary Fig. 2 (Additional File 3). For consistency and comparability reasons, we defined statistical descriptions for the POLAR_MI population, for the modified POLAR_MI population and for each population of the five research projects (Supplementary Fig. 2, Additional File 3). While these general, informative descriptions were consistently calculated for all POLAR_MI analyses, each research project complemented them with its project-specific descriptions to make the result interpretation feasible (see [9, 10] for applications). Details on adaptations and refinements of this approach to be applicable to the CDS data within the MII are provided in Supplementary Methods (Additional File 4) [44–47].

To demonstrate the feasibility of our multicentre approach, we provide the meta-analysed description of both the POLAR_MI population and the modified POLAR_MI population (Supplementary Fig. 2, Additional File 3) according to the above-mentioned specifications. These populations are consistent across all POLAR_MI research projects [9, 10]. Furthermore, relying on the latter population, we provide information on the availability of medication data in general – overall and stratified by the classification system. Here, we need to distinguish between the Anatomical Therapeutic Chemical (ATC) code [44] and the German Pharmazentralnummer (PZN; pharmaceutical central number) [48].

For the random-effects meta-analysis, we used R (version 4.2.2) and the R packages *meta* (version 6.0.0) [49] and *metamedian* (version 0.1.5) [50, 51]. Meta-analysis results are provided as median and proportion, respectively, with 95% confidence interval (CI). These numbers are accompanied by the number of encounters with information on the respective characteristic.

## Results

First, we summarise our lessons learned during local statistical analysis and central meta-analysis when performing this multicentre project with the proposed distributed analysis approach. Finally, we provide a real data application from POLAR_MI. For additional information on methodological aspects, we also refer to the POLAR_MI-associated investigation [10].

### Local statistical analysis at each data integration centre

#### The data snapshot

The decision to perform all analyses at a DIC on the same previously taken data snapshot (Fig. 1) was based on observations during the first retrieval and analysis runs, as it turned out that downloading the data from the FHIR server during the retrieval part was very time-consuming. Besides increasing efficiency, we realised better consistency between the defined analyses within POLAR_MI and enabled the replicability of each analysis. Finally, the data snapshot enabled us to redo the analyses even if the FHIR resources changed due to updated profiles (during POLAR_MI: CDS version 1.0).

#### Referencing between FHIR resources

The way of referencing between different FHIR resources varied between the DIC. Although the resources Patient and Encounter are introduced as equivalent central units, according to the CDS definition, each resource must provide a reference to the patient, whereas a reference to the encounter is optional. In the latter case, if a resource lacks a reference to the encounter, this reference must be inferred. In this situation, we decided to link this resource via temporal overlap information to the encounter. This means that the resources of a patient were linked to a specific encounter of the patient, if the time stamp of the resource was within the time interval of the hospital stay of the respective encounter (defined by the encounter’s start and end date). If resources could not be linked to an encounter, they were not included in the analysis, introducing missing information on the respective item. Furthermore, we excluded encounters of a patient, if they exhibited a time overlap, because, then, the hospital stay (with related resources) could not be defined uniquely (Supplementary Table 2, Additional File 2).

#### Integration of available time stamps

Ideally, from a naïve researchers’ perspective, each time stamp (encoded in an “effectiveDateTime*”* or similar element of a FHIR resource) would have been documented in real-time and corresponded to the actual time of the event. However, this concept did not match the common practice of post-documentation (in the HIS) found with our collaboration partners (Supplementary Table 4, Additional File 2). This handling of time stamps also caused problems when considering medication-related adverse events. It was often impossible to identify the sequence of medication and (potential) adverse event based on the FHIR data. Consequently, associations found in the EMR data had to be interpreted with caution, as shown, for example, in the POLAR_MI-associated work [10].

#### Integration of available medication resources

Within POLAR_MI, all research questions were related to medications. To extract this information, we had to define proxy variables for “documented medication”, “ambulant medication” and “discharge recommendation”, because not all required information was provided by our collaborating partners during this study in a machine-readable form (Supplementary Table 5, Additional File 2). Furthermore, we required information on specific medications. Hence, we decided that only encounters with at least one documented 7-character ATC code in a MedicationStatement or a MedicationAdministration resource (both resources treated as indistinguishable (reasons: Supplementary Table 5, Additional File 2), although expressing semantically different concepts) were included in the analysis and, consequently, defined the additional inclusion criteria for all research projects within POLAR_MI (Supplementary Fig. 2, Additional File 3). The MedicationRequest resource, which might have facilitated the assessment of documented medications, was not DIC-wide available at the time of analysis, because the balloting process within the MII was still running. Finally, we did not include any medication query that considered (daily) dosages, because we could not reliably extract dosage information from the FHIR data. The main reason was that the CDS instructions for ingredient integration allowed a broad variety of valid, but very distinct solutions to map ingredients to drugs. This mapping might focus on the most important ingredient or active ingredients only, but any other solution was possible as long as the ingredient list consisted of at least one item.

#### Laboratory values

A specific laboratory value can be identified via LOINC codes (Logical Observation Identifiers Names and Codes) [52, 53]. A drawback in LOINC analysis is, that for one parameter (e.g., creatinine) a multitude of LOINC mapping options may exist and the selection of the most appropriate LOINC code(s) is subject to the laboratory facilities of the hospitals (e.g., depending on SI unit, liquid-compartment and method). As a consequence, centres did not provide all LOINC codes for each laboratory value. Hence, researchers had to check prior to their data analysis whether the required LOINC code(s) were available at the centres participating in the analyses and, if necessary and possible, adapted their LOINC code list. Otherwise, the proportion of encounters with missing information on this laboratory value increased. Units were an additional issue. For each LOINC code, there is a recommended, but not mandatory unit. In the CDS, there were two ways of providing units: (i) a machine-readable or (ii) a human-readable form of the unit. It was optional for each DIC to integrate units in one or both forms. Consequently, we found substantial variability in unit specifications, which is in line with [54]. Differences arose from the usage of both upper and lower cases (e.g., “ml” versus “mL”), of space between the operators or of the multiplicative inverse (e.g., “mg/dL” versus “mg / dL” versus “mg dL^− 1^”). Finally, the metric value did not always fit the provided unit, which we often could identify during our analysis and then provided this feedback to the respective DIC. Our approaches, which were developed in order to cope with the heterogeneity of laboratory values in the FHIR data, both among different DIC and within a single DIC, are provided in Supplementary Table 6 (Additional File 2).

#### Diagnoses

In general, available diagnoses were always interpreted as “documented”. If at least one diagnosis was documented for an encounter, not-mentioned diagnoses were taken as “not documented”. Information on diagnoses was missing for an encounter, if no diagnosis was documented for this encounter. When relying on data (and their time stamps) generated in compliance with the § 21 German Hospital Reimbursement Act, a distinction between admission diagnoses, comorbidity diagnoses and diagnoses identified during the stay was not possible (see also result subsection on integration of time stamps). As some DIC integrated this kind of data, we integrated all diagnoses available in the FHIR data into the analysis, regardless of their type.

#### A priori data item selection (for data reduction) by some data integration centres

In some DIC, the modules of the locally executed part of the POLAR_MI ETL Pipeline did not run on the complete FHIR database but on a subset tailored to the medical data that was required for the POLAR_MI analyses. There were several reasons for subset building. The development of the ETL pipeline required a stable and comprehensible data collection while the DIC itself were still under construction. Furthermore, the DIC saved local IT resources including reduced analysis execution time and prevented their main FHIR server from unintended server shutdown accidently induced by the ETL pipeline. Additionally, this procedure avoided local data protection issues, as it could be guaranteed that the ETL pipeline processed only data as agreed by the consortia. Within POLAR_MI, it was appropriate to narrow down (i) encounters and other FHIR resources to the analysis interval and (ii) laboratory values to evaluated/analysed LOINC codes without encounter exclusion. In contrast, filtering encounters/patients for specific medications or diagnoses beyond the defined in- and exclusion criteria had to be prevented, because this would have made prevalence estimation and association analysis impossible.

#### Handling missing data

Some of the assumptions and adaptions described in the previous sections were necessary to overcome issues related to missing data. Possible reasons for missing data were the following:

- FHIR resource was missing, not readable or empty (e.g., related to medication, laboratory value, diagnosis, encounter or patient). Possible reasons comprised the following: (i) wards not connected to the FHIR server, (ii) clinical source systems not connected to the FHIR server and (iii) data not integrated (if the item was not mandatory) or integrable by the DIC (due to readability/interoperability).
- Information was already missing in the EMR.
- Missing value was induced by the POLAR_MI ETL Pipeline.
- Encounter reference was missing in the resource.

Related to inclusion criteria, encounters with missing required information (patient’s age, 7-character ATC code, information on whether it was an inpatient stay) were excluded from the analysis. There were no further POLAR_MI-wide agreements for handling missing data, but each research project required additional solutions to deal with missing information. For each decision, advantages (mainly increased sample size, i.e., more contributing centres and more analysable encounters per centre) and risks (especially the introduction of bias) were weighed against each other. When missing information did not lead to the exclusion of an encounter, the number of encounters with missing information on the specific variable was counted during analysis.

#### Technical realisation of the POLAR_MI ETL pipeline

As each DIC and its IT infrastructure (including HIS and CDS) were unique (including the interpretation and realisation of MII specifications), the retrieval module had to be partly adapted to local settings and characteristics. The heterogeneity of FHIR servers was so substantial that setups working well at one DIC completely failed in another. For instance, we provided an option to “download all encounters in one request” as an alternative to the default “download encounters monthly”. Whereas the default parameter overcame cache and pagination issues, its alternative avoided redundancy from date specification issues. Further server-specific issues comprised run-time, early expiration of the request token and early termination of a token-free request. Thus, the implementation of the retrieval module was a challenging, iterative process, for which we utilised clearly defined development cycles (Supplementary Fig. 3, Additional File 3). In the end, the POLAR_MI ETL Pipeline was a composition of the least common denominator, because we were unable to identify an appropriate, robust parameter setting that fitted all server needs in one and balanced the server workload at the same time.

### Central meta-analysis of local results

For reproducibility, consistency and transparency, we developed a checklist to inspect the local analysis results and to support the decision for both inclusion in the meta-analysis and necessity of subgroup analyses. Items comprised the (i) availability/completeness of results files, (ii) occurrence of errors or warnings during local analysis, (iii) date of the data snapshot creation for consistency across different analyses, (iv) inclusion criteria check, (v) consistency of absolute frequencies, (vi) plausibility of proportions and (vii) numerical/convergence issues in case of regression modelling. The meta-analysis result checks also targeted heterogeneity and suspicious local results indicating data or module/script issues that might be solvable by local adaptations. All the above-mentioned decisions were made in collaboration with pharmacologists, medical biometricians and computer scientists after consultation with the respective DIC.

### POLAR_MI real data application

Twelve participating centres were able to transmit local results. Due to local data protection requirements, two centres (Munich, Tübingen) could not be included in the meta-analysis. Consequently, ten centres (Bonn, Erlangen, Freiburg/Breisgau, Gießen, Halle/Saale, Hamburg, Heidelberg, Jena, Kiel and Leipzig) were included in the final meta-analysis. In about half of them, the complete (predefined) POLAR_MI analysis time interval was supported (four times about 4.0 years, once about 3.5 years, twice about 2.5 years, twice about 1.5 years and once about 1.0 year). We took local data snapshots between April 2023 and January 2024 and ran local statistical analyses in December 2023 and January 2024.

#### The POLAR_MI population description

The POLAR_MI population (Supplementary Fig. 2, Additional File 3) comprised in total 788,127 encounters from 501,613 patients. On the encounter-level, the median age (95% CI) was 60.1 (58.1, 62.1) years, 49.5% (47.7%, 51.2%) were female and the median length of stay was 3.0 (2.2, 3.8) days. Information on diagnoses was available for about 98.6% (90.0%, 99.8%) of the encounters. Among them, the median number of different diagnoses per encounter was 6.8 (6.2, 7.4) leading to a median Charlson comorbidity index of 2.5 (2.2, 2.8) points. Regarding the availability of 7-character ATC codes, this medication information was available for 63.7% (31.9%, 86.8%) of the encounters with a median number of documented, different 7-character ATC codes of 6.6 (5.1, 8.2) per encounter. Further details including stratification for age categories and sex are provided in Supplementary Table 7 (Additional File 2). The difference between the availability of diagnoses and of medications could (partly) be explained by the data origin. While information like diagnoses required due to § 21 of the German Hospital Reimbursement Act was available for the majority of encounters, medication information was only accessible for wards, which documented medication electronically and were already connected to the FHIR server for data transfer. We will elaborate more on medication data availability in the subsequent section on the medication coding system.

#### Technical exclusion reasons

**Fig. 2:**
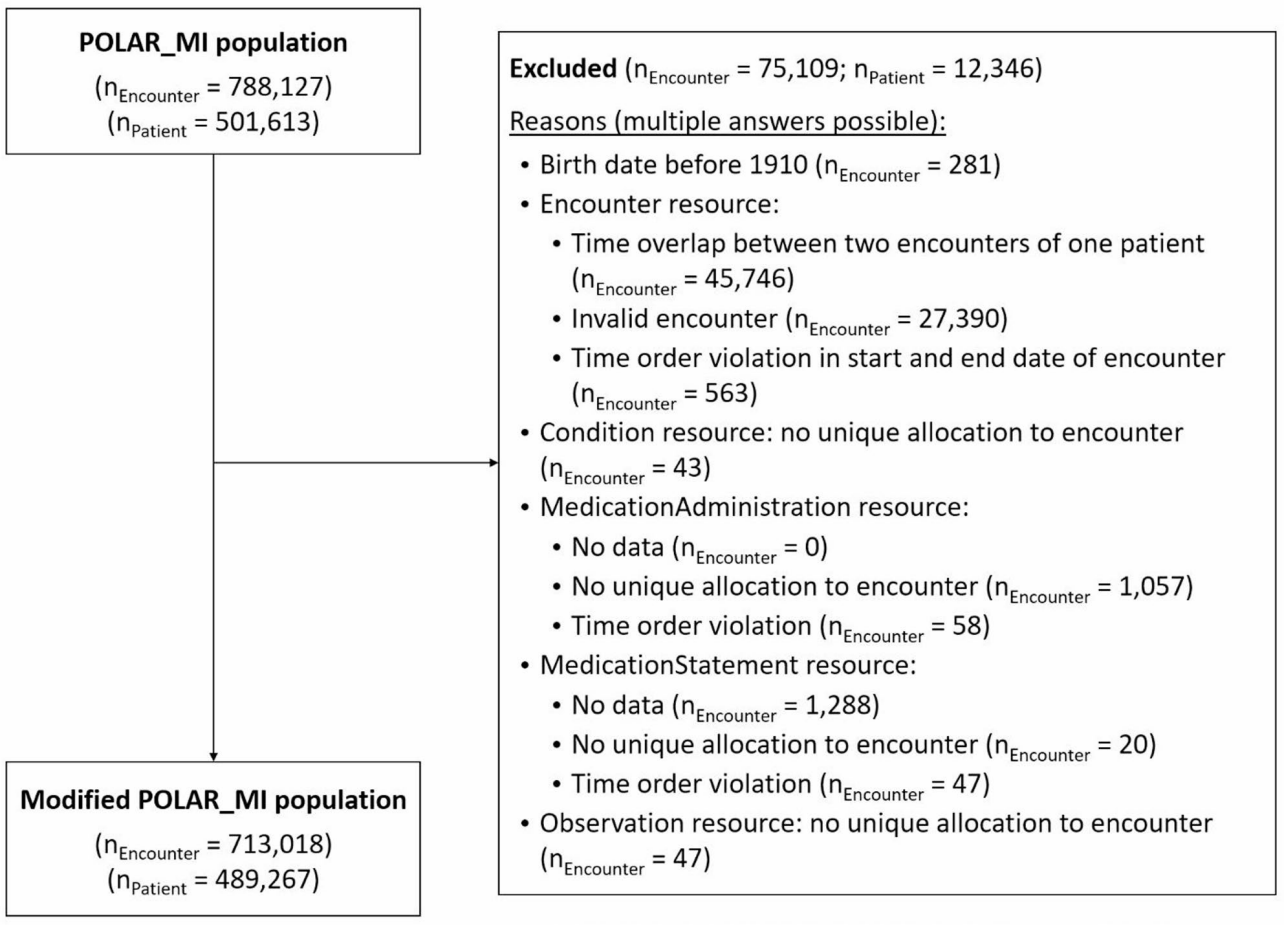
Flowchart. The number of both encounters (n_Encounter_) and patients (n_Patient_) that were included in and excluded from, respectively, the meta-analysis across the ten centres is provided for the POLAR_MI population and the modified POLAR_MI population, i.e., the POLAR_MI population reduced by encounters excluded for technical reasons (see Supplementary Table 7, Additional File 2). For the excluded encounters, the frequency of the exclusion reasons is provided. Note, that some centres provided MedicationAdministration resources, while others only provided MedicationStatement resources. In our analysis, an encounter is equivalent to a case, i.e., a hospital stay of a patient

Excluding encounters fulfilling technical exclusion criteria from the POLAR_MI population reduced the population size to 713,018 encounters from 489,267 patients. Among encounters, the main reason for exclusion (45,746 out of 75,109 excluded encounters) was a time overlap between two encounters of a patient. Notably, the exclusion of one encounter resulted in the exclusion of the entire patient, which avoided, for instance, bias from time course issues (e.g., hospital re-admission). Further details on the frequency of exclusion reasons are given in the flowchart (Fig. 2). These exclusions led to small shifts in the population description (Supplementary Table 7, Additional File 2).

#### Medication data availability (related to medication coding systems)

Among encounters in the modified POLAR_MI population, about one third of the encounters (32.5% with a 95% CI of (10.2%, 67.1%)) lacked medication resources (Supplementary Table 8, Additional File 2). The heterogeneity across centres was large, as indicated by the range of the proportion of encounters with available medication resources across the DIC (range 10%-100%). If medication resources were linked to an encounter, then both ATC code and German PZN were regularly provided (99.9% (99.5%, 100.0%), range 97%-100%), usually even as 7-character ATC code and 8-digit PZN (99.6% (98.2%, 99.9%), range 84%-100%). As indicated by the ranges, the heterogeneity across the centres in providing this information was substantial. In general, ATC codes with less than seven characters are allowed, but these ATC codes always specify a drug group instead of a specific medication. In our data, the probability that there was at least one 7-character ATC code, if any ATC code was documented for an encounter, was about 99% to 100% for all centres. In contrast, the leading zero to translate the former 7- to the currently valid 8-digit PZN was mostly, but not completely implemented at all DIC at the time of our analyses. The proportion of encounters with 7-digit PZN among encounters with any PZN varied mostly between 0% and 1% with a maximum of about 15%. This also affected the availability of 8-digit PZN in case of an available 7-character ATC code. Here, the proportion reached a minimum at about 85%, but was above 97% otherwise. Based on these and previous observations, we introduced the above-mentioned inclusion criterion, that an encounter had to have at least one documented 7-character ATC code. Applying this criterion to the modified POLAR_MI population, 365,344 encounters in 270,384 patients remained in the population (Supplementary Table 9, Additional File 2).

## Discussion

The cooperative project POLAR_MI aimed at detecting medication-related risks attributed to polymedication in adult patients from German university hospitals. Here we reported that such a large-scale, multicentre approach with ten German university hospitals and about half a million patients is possible despite very heterogeneous HIS infrastructure. Data from routine healthcare was analysed via the local DIC established by the MII and applying the POLAR_MI ETL Pipeline, comprising all parts of the developed distributed analysis approach that preserved privacy. The success was possible due to the harmonised and interoperable CDS. However, it was necessary to overcome a series of difficulties.

### Missing, incomplete and heterogeneous data – sources and implications

The specifications of the CDS are strict in terms of required formats, but – from the perspective of a data researcher – relatively flexible in the guidelines for filling the items of the CDS [6]. Although FHIR allows specifying a “data absent reason” instead of using default values for missing information (for, e.g., patient’s birthdate), this information was hardly ever provided at the time of our analyses. Furthermore, some items such as the discharge date were kept optional, and, consequently, remained empty for various reasons. Besides information that was already missing in the HIS, missing information could also be introduced by lacking links between different FHIR resources for an encounter or a patient. These aspects are covered by our technical exclusion reasons. Some of these reasons like the time overlap of several hospital stays of a patient and, in parts, the reliability of available time stamps might be overcome by more precise primary documentation in the hospital. However, the content of FHIR databases was heterogeneous across the DIC [6] and, for various data, it remained unclear where to find which information and whether the desired information was indeed found.

In general, handling missing information is essential, but the introduction of bias (and errors) cannot be ruled out and their impact on the analysis results is so far unclear [55]. Some of the missing information might be missing (completely) at random but others not. Within POLAR_MI, we were especially concerned about the latter, because not all wards (or even not all ward’s IT documentation systems) were connected to the FHIR server at some DIC [6]. This had an impact on the case-mix (i.e., the distribution of disease severity among the encounters/patients) and in parts on the availability of medication information. Consequently, all assumptions, definitions, deduced phenotypes with related sources and underlying populations must be considered [56] and clearly described [57]. Only including encounters/patients with a comprehensive EMR or patients with several stays might result in a bias towards sicker patients [58, 59]. For instance, the determination of some specific laboratory measurements depends on the disease (severity), the medical discipline or whether a (specific) pathology is suspected. Of note, despite harmonisation efforts, not all values (e.g., laboratory values) are always reliably comparable [6] and results must be interpreted cautiously [60].

### Establishing congruence between the research question and available data

The use-case POLAR_MI started in a phase in 2018, when the implementation of the CDS was just initiated, so that we had to work with data (structures) under construction [3, 6]. Our lack of experience in the beginning regarding the extent of CDS flexibility prevented us from preparing a DIC-typical, unideal test data set prior to module development to advance this development. Hence, any coding had to rely on an iterative process with trial and error between DIC and the multidisciplinary analysis team (Supplementary Fig. 3, Additional File 3). As by-product, we could provide individual feedback to the DIC. This feedback dealt with data (curation) issues and partly enabled improvements in the local and global CDS implementations, e.g., the consequent implementation of the 8-digit PZN in several DIC. Of course, the multidisciplinary analysis team also benefited from the feedback provided by the DIC. A systematic investigation of implausible relations and suspicious values as well as the systematic ascertainment of possibly observable value combinations improved the module handling itself. For instance, considering diagnosis, cases with so-called “double diabetes”, i.e., individuals suffering from both diabetes mellitus type 1 and type 2, are rare observations, but were observable (and proven as non-erroneous documentation) in our analyses [61]. Due to our systematic ascertainment, we had already implemented handling of cases with double diabetes prior to the first identification of such an encounter in the data. Here, the module was prepared to handle this rare finding instead of discarding it. We conclude that both DIC and multidisciplinary analysis teams need to understand both the technical characteristics of the data and their clinical relevance, which was also highlighted by Brat and colleagues [62] and Kohane and colleagues [57]. A crucial matter of DIC is to improve the data integration process and reduce human intervention during data transfer [6]. Hereby, the POLAR_MI ETL Pipeline generated abundant and welcome feedback to the DIC about data quality issues presumably caused by mapping HIS annotation to CDS specification.

### Observations within a distributed analysis approach for patient-level electronic medical records data

Analyses of routine healthcare data across many institutions (and countries) with heterogeneous HIS infrastructure exhibit challenges, which were also observed in a similar distributed analysis approach on non-CDS routine healthcare data. Moal and colleagues [63] implemented a similar distributed analysis approach to analyse acute respiratory distress syndrome after a SARS-CoV-2 infection. There, they used patient-level EMR from healthcare systems in Europe and the United States of America. While the analyses within POLAR_MI relied on data according to the CDS specifications unifying the format for HIS data integration, Moal and colleagues [63] used a pre-specified format for data extraction of pre-specified items/variables/measures/values from the EMR. Data extraction was locally implemented [62]. The extracted data was then locally analysed and, after several quality controls, the local results presented as count data (no data on patient-level) were combined in a central, random-effects meta-analysis [62, 63]. They also largely relied on the diagnoses based on ICD-10 codes (International Statistical Classification of Diseases and Related Health Problems, 10th Revision [64]) from the billing code system. However, Moal and colleagues [63] only used the first three digits from these codes to address differences in the coding routine across participating hospitals; this might have introduced a bias and missing data [63]. To overcome issues related to laboratory values, they focussed on five selected values only and nevertheless observed large heterogeneity within centres, between centres and between countries, which might also be linked in parts to differences in laboratory and clinical routines [62]. Furthermore, mappings from the laboratory values to the LOINC codes were often missing and an individual, local interpretation of the laboratory value was necessary. Brat and colleagues [62] suggested unit conversion and called for reference ranges of the laboratory values. The observations related to heterogeneity and mapping are in line with our experiences. Additionally, coding and spelling errors (ATC codes, LOINC codes, units) seem to be a severe issue, as their identification is challenging to impossible, especially in distributed analyses.

### Required prerequisites for the integration of laboratory values

Laboratory values are variables of importance to many biomedical research projects which is why we decided to put a special focus on our observations regarding their integration. The first harmonisation step for their integration is the mapping of laboratory value identifier to the appropriate LOINC code (e.g., “1988-5” for “C-reactive protein [Mass/volume] in Serum or Plasma”) and the translation of units into UCUM-conform (UCUM: Unified Code for Units of Measure), machine-readable units (e.g., “G/L” to “10^9/L”) [65, 66]. This step requires familiarity with local laboratory data and knowledge of both coding systems. In the second harmonisation step for reliable integration of laboratory values from different centres into statistical analyses, different identifiers for similar values have to be grouped (e.g., “1988-5” for “C reactive protein [Mass/volume] in Serum or Plasma” and “105126-7” for "C reactive protein [Mass/volume] in Serum, Plasma or Blood“) and values with different units have to be converted (e.g., 1 g/L = 1000 mg/L).

LOINC coding and UCUM-conform transformation can only be done sufficiently at the local centres, but would benefit from central recommendations that can help to reduce inconsistencies between centres and facilitate laboratory value integration in multicentre analyses. Validating UCUM conformity can be done using tools like the UCUM Software Library [67]. A more comprehensive solution would be to integrate LOINC and UCUM coding into primary laboratory information systems, potentially requiring legislation. As both LOINC code grouping and common unit selection may depend on the research question, a more centralised approach might here be more appropriate to cover these issues within a multicentre (distributed) analysis. However, researchers currently lack comprehensive solutions for this and must create mapping tables themselves.

### Stability of FHIR requests

Considering issues caused by the POLAR_MI ETL Pipeline, we observed that even for identical queries applied within the ETL pipeline to the same state of data elements, i.e., fixed data in the same DIC, the final data snapshots slightly differed. While some of these differences could be traced back to a failed load balancing, others could be narrowed down to resource conflicts in a non-thread-safe library, when parallelisation of FHIR search requests was done by threading. This issue could be avoided by parallelisation of FHIR search requests via separate processes. Unfortunately, this solution came up late in the project’s time, such that the data extraction could not be re-run with the respective patch. Due to limited resources, we cannot provide evidence here, whether other circumstances (e.g., providing the FHIR servers as shared resources and not exclusive to POLAR_MI) also contributed to the instability of FHIR requests.

### Collaborations and professional, multidisciplinary exchange induced by POLAR_MI

Through the interdisciplinary exchange between the DIC and the POLAR_MI analysis team during the joint ETL pipeline development, various technical developments and ad hoc working groups were introduced at the DIC level. The central role of the DIC in medication-related improvements at the MII level was identified. Feedback loops required for implementing the ETL pipeline also provided external evaluation of the DIC’s data integration level, helping to identify and to resolve systemic data integration issues. Given the diverse infrastructure landscape across German university hospitals, the following initiatives were undertaken to standardise medication information. Firstly, feedback from POLAR_MI raised awareness at the DIC for distributed analysis prerequisites and specific research questions, fostering FHIR interface development for medication data integration. Secondly, a free special license of the MMI Pharmindex [68] was offered to MII sites to enrich FHIR resources with additional medication information, supporting standardised and CDS-conform provision of pharmaceutical information. Thirdly, efforts within POLAR_MI also impacted CDS developments, including participation in CDS meetings and documentation differentiation of outpatient and inpatient medication, released in CDS version 2.0 in 2023 [69].

### Summary of major challenges and ideas for future resolution

First, we found a large heterogeneity in the received local results, which was related to the reported numbers of patients, encounters, medications, diagnoses and missing values. Several factors contributed to this heterogeneity. Identified reasons were that local HIS systems were not mapped fully to the FHIR standard at all DIC, that the interpretation of CDS specifications varied among DIC and that resources to execute the ETL pipeline were partially limited. Ongoing efforts to improve the situation include spreading implementation of the (revised) CDS specifications, increasing manpower at the DIC and enhancing robust and operational readiness on distributed analysis approaches. Researchers can now apply for (distributed) analyses of the CDS data as a multicentre study, e.g., via the German Portal for Medical Research Data (*Deutsches Forschungsdatenportal für Gesundheit*, FDPG) [70].

Second, time stamps for diagnoses posed a problem as patients’ diagnoses are often coded post-discharge without indicating when they were made. This can lead to misleading results when, e.g., linking medications to disease-related outcomes. To address the sequence of timings in the future, this information must be expanded into the FHIR server at the DIC by filling in properly the corresponding resource, together with an encounter reference in each item and a reliable time stamp. This probably necessitates stakeholder discussions.

Third, we identified missing values on many levels and treated them within POLAR_MI as if they were missing (completely) at random. As addressing data quality remains crucial, we plan to undertake a rigorous missing data analysis following well-established techniques [71] to finally improve data quality and achieve interpretable prevalence estimates for selected potentially inappropriate medications.

Fourth, the iterative, repeated, distributed ETL process lasted much longer than expected. Large-scale data analysis without direct data access was challenging due to the level of data heterogeneity and the newly implemented CDS. Thus, more direct data analysis processes for distributed or federated computation are needed to efficiently leverage the potential of distributed data analyses in the future, e.g., also via DataSHIELD [72] or Personal Health Train [73]. An alternative to the current MII specifications applied at the DIC might be the OMOP (Observational Medical Outcomes Partnership) common data model from the OHDSI (Observational Health Data Sciences and Informatics) consortium, which is an open community data standard, which is tailored to provide observational data for efficient analyses [74].

## Conclusions

Within POLAR_MI, we successfully conducted one of Germany’s largest data-analysis efforts on routine healthcare data from ten university hospitals focusing on medication safety. We developed an ETL pipeline for the CDS FHIR profiles and adapted it for each centre’s source systems. Despite obstacles such as data mapping issues, data incompleteness, time stamp issues and (population) heterogeneity, we managed to address many of these with a set of transparent simplifications and assumptions. Given the ongoing progress of the MII, it is important to repeat our investigations on more recent data to monitor improvements in data completeness and quality as we plan to do within the ongoing INTERPOLAR (INTERventional POLypharmacy - drug interActions - Risks) project [75]. Finally, the rationale behind our developed ETL pipeline including the identified challenges and proposed solutions to address these challenges within a distributed analysis approach can be adapted to similar initiatives in other countries or healthcare systems, if an equivalent to the DIC and the CDS are available – within both distributed/federated and central analysis approaches for data from routine healthcare.

## Supplementary Information

Below is the link to the electronic supplementary material.

Supplementary Material 1: Additional File 1: Membership list of POLAR_MI (PDF)

Supplementary Material 2: Additional File 2: Supplementary Tables (PDF)

Supplementary Material 3: Additional File 3: Supplementary Figures (PDF)

Supplementary Material 4: Additional File 4: Supplementary Methods (PDF)

Supplementary Material 5: Additional File 5: Ethics committees and the reference numbers of the participating centres

## Data Availability

The initial, local data snapshots containing the centres’ pseudonymised patient data, i.e. the data from the patients’ electronic medical records from routine inpatient care, cannot be shared, as the General Data Protection Regulation prohibits their transfer to third parties without patient consent. However, researchers can apply for the data and related analysis via the German Portal for Medical Research Data (*Deutsches Forschungsdatenportal für Gesundheit*, FDPG; https://forschen-fuer-gesundheit.de). All developed software components including the R scripts are available via the ‘Health Atlas - Local Data Hub/Leipzig’ at https://www.health-atlas.de/lha/83F28HCE9N-6. The authors confirm that the complete meta-analysis results of this study are provided in this article and its supplementary materials.

## Abbreviations

ADE: Adverse drug event(s)
ATC: Anatomical Therapeutic Chemical
CDS: Core data set
CI: Confidence interval
DIC: Data integration centre(s)
eGFR: Estimated glomerular filtration rate
EMR: Electronic medical record(s)
ETL: Extract, transform and load data
FDPG: Deutsches Forschungsdatenportal für Gesundheit (German Portal for Medical Research Data)
FHIR: Fast Health Interoperability Resources
HIS: Hospital information system(s)
HL7: Health Level Seven
ICD-10: International Statistical Classification of Diseases and Related Health Problems (10th Revision)
ICD-10-GM: German modification of ICD-10
INTERPOLAR: INTERventional POLypharmacy - drug interActions - Risks
IT: Information technology
LOINC: Logical Observation Identifiers Names and Codes
MII: Medical Informatics Initiative Germany
OHDSI: Observational Health Data Sciences and Informatics
OMOP: Observational Medical Outcomes Partnership
POLAR_MI: POLypharmacy, drug interActions and Risks
PZN: Pharmazentralnummer (German pharmaceutical central number)
SAP: Statistical analysis plan
SI: Système international d’unités (international system of units)
UCUM: Unified Code for Units of Measure

## References

[1] Statistisches Bundesamt. Grunddaten der Krankenhäuser 2020. Fachserie 12 / Reihe 6.1.1. Wiesbaden, Germany 2024.

[2] Semler SC, Wissing F, Heyder R. German Medical Informatics Initiative. Methods Inf Med. 2018;57(S 01):e50–6. 30016818 10.3414/ME18-03-0003 PMC6178199

[3] Albashiti F, Thasler R, Wendt T, Bathelt F, Reinecke I, Schreiweis B. Die Datenintegrationszentren – Von der Konzeption in der Medizininformatik-Initiative zur lokalen Umsetzung in einem Netzwerk Universitätsmedizin. Bundesgesundheitsblatt Gesundheitsforschung Gesundheitsschutz. 2024;67(6):629–36. 38662020 10.1007/s00103-024-03879-5 PMC11166806

[4] Health Level Seven International. HL7^®^, HEALTH LEVEL SEVEN^®^, FHIR^®^ and the FHIR ^®^ [registered with the United States Patent and Trademark Office]. Available from: http://hl7.org/fhir/.

[5] Kamdje-Wabo G, Gradinger T, Lobe M, Lodahl R, Seuchter SA, Sax U, Ganslandt T. Towards Structured Data Quality Assessment in the German Medical Informatics Initiative: Initial Approach in the MII Demonstrator Study. Stud Health Technol Inform. 2019;264:1508–9. 31438205 10.3233/SHTI190508

[6] Ammon D, Kurscheidt M, Buckow K, Kirsten T, Lobe M, Meineke F, et al. Arbeitsgruppe Interoperabilität: Kerndatensatz und Informationssysteme für Integration und Austausch von Daten in der Medizininformatik-Initiative. Bundesgesundheitsblatt Gesundheitsforschung Gesundheitsschutz. 2024;67(6):656–67. 38753022 10.1007/s00103-024-03888-4 PMC11166738

[7] Scherag A, Andrikyan W, Dreischulte T, Dürr P, Fromm MF, Gewehr J, et al. POLAR – “POLypharmazie, Arzneimittelwechselwirkungen und Risiken” – wie können Daten aus der stationären Krankenversorgung zur Beurteilung beitragen? Prävention und Gesundheitsförderung. 2022.

[8] Mosshammer D, Haumann H, Morike K, Joos S. Polypharmacy-an Upward Trend with Unpredictable Effects. Deutsches Ärzteblatt International. 2016;113(38):627–33. 27743469 10.3238/arztebl.2016.0627 PMC5078862

[9] Redeker L, Kesselmeier M, Mussawy B, Grabe S, Rottenkolber M, Thalheim T, et al. Use of potentially inappropriate medication and association with falls during hospitalisation: an analysis based on electronic health records (POLAR_MI project). Drugs - Real World Outcomes. 2026;13:15–27. 41258957 10.1007/s40801-025-00528-4 PMC13003043

[10] Wermund AM, Thalheim T, Medek A, Schmidt F, Peschel T, Strübing A, et al. Challenges in detecting and predicting adverse drug events via distributed analysis of electronic health record data from German university hospitals. PLOS Digit Health. 2025;4(6):e0000892. 40569944 10.1371/journal.pdig.0000892 PMC12200832

[11] Medizinformatik-Initiative. Kerndatensatz Modul Person 2023 [Accessed 2024/11/20]. Available from: https://www.medizininformatik-initiative.de/Kerndatensatz/Modul_Person/IGMIIKDSModulPerson.html.

[12] Medizinformatik-Initiative. Kerndatensatz Modul FALL 2021 [Accessed 2024/11/20]. Available from: https://www.medizininformatik-initiative.de/Kerndatensatz/Modul_Fall/IGMIIKDSModulFall.html.

[13] Medizinformatik-Initiative. Kerndatensatz Modul Diagnose 2021 [Accessed 2024/11/20]. Available from: https://www.medizininformatik-initiative.de/Kerndatensatz/Modul_Diagnose/IGMIIKDSModulDiagnose.html.

[14] Medizinformatik-Initiative. Kerndatensatz Modul Medikation 2022 [Accessed 2024/11/20]. Available from: https://www.medizininformatik-initiative.de/Kerndatensatz/Modul_Medikation/ImplementationGuide-1.x-IGMIIKDSModulMedikation-1.x.html.

[15] Medizinformatik-Initiative. Kerndatensatz Modul Laborbefund 2021 [Accessed 2024/11/20]. Available from: https://www.medizininformatik-initiative.de/Kerndatensatz/Modul_Laborbefund/IGMIIKDSModulLaborbefund.html.

[16] GitLab Inc. GitLab [GITLAB is a trademark of GitLab Inc. in the United States and other countries and regions]. Available from: https://about.gitlab.com/.

[17] Merkel D. Docker: lightweight linux containers for consistent development and deployment. Linux J. 2014;2014(239):2.

[18] Docker Inc. Docker^®^ [registered in the United States and/or other countries]. Available from: https://www.docker.com/.

[19] Docker Inc. dockerdocs [Accessed 2024/11/20]. Available from: https://docs.docker.com/.

[20] Barrett T, Dowle M, Srinivasan A, Gorecki J, Chirico M, Hocking T. data.table: Extension of `data.frame` [R package]. Available from: https://CRAN.R-project.org/package=data.table

[21] Wickham H, François R, Henry L, Müller K, Vaughan D. dplyr: a grammar of data manipulation [R package]. Available from: https://CRAN.R-project.org/package=dplyr.

[22] Palm J, Meineke FA, Przybilla J, Peschel T. "fhircrackr": An R Package Unlocking Fast Healthcare Interoperability Resources for Statistical Analysis. Appl Clin Inf. 2023;14(1):54–64. 10.1055/s-0042-1760436 PMC9876659 36696915

[23] Wickham H. ggplot2: Elegant Graphics for Data Analysis. New York, USA: Springer; 2016.

[24] Wickham H, Bryan J. readxl: Read excel files [R package]. Available from: https://CRAN.R-project.org/package=readxl.

[25] Wickham H, Hester J, Ooms J. xml2: Parse XML [R package]. Available from: https://CRAN.R-project.org/package=xml2.

[26] McLeod A, Xu C, Lai Y. bestglm: Best subset GLM and regression utilities [R package]. Available from: https://CRAN.R-project.org/package=bestglm.

[27] Signorell A. DescTools: Tools for descriptive statistics [R package]. Available from: https://CRAN.R-project.org/package=DescTools.

[28] Warnes G, Bolker B, Lumley T, Magnusson A, Venables B, Ryodan G, Moeller S. gtools: various R programming tools [R package]. Available from: https://CRAN.R-project.org/package=gtools.

[29] Lumley T, Miller A. leaps: regression subset selection [R package]. Available from: https://CRAN.R-project.org/package=leaps.

[30] Zeileis A, Hothorn T. Diagnostic Checking in Regression Relationships. R News. 2002;2(3):7–10.

[31] Grolemund G, Wickham H. Dates and Times Made Easy with lubridate. J Stat Softw. 2011;40(3):1–25.

[32] van Buuren S, Groothuis-Oudshoorn K. mice: Multivariate Imputation by Chained Equations in R. J Stat Softw. 2011;45(3):1–67.

[33] Schratz P. R package ‘oddsratio’: Odds ratio calculation for GAM(M)s & GLM(M)s [R package]. Available from: https://CRAN.R-project.org/package=oddsratio.

[34] Liaw A, Wiener M. Classification and Regression by randomForest. R News. 2002;2(3):18–22.

[35] Petrie A. regclass: tools for an introductory class in regression and modeling [R package]. Available from: https://CRAN.R-project.org/package=regclass.

[36] Maechler M, Rousseeuw P, Croux C, Todorov V, Ruckstuhl A, Salibian-Barrera M, et al. robustbase: basic robust statistics [R package]. Available from: http://CRAN.R-project.org/package=robustbase.

[37] Khan MRA, Brandenburger T. ROCit: Performance assessment of binary classifier with visualization [R package]. Available from: https://CRAN.R-project.org/package=ROCit.

[38] Therneau T, Atkinson B. rpart: Recursive partitioning and regression trees [R package]. Available from: https://CRAN.R-project.org/package=rpart.

[39] Yee TW. Vector Generalized Linear and Additive Models: With an Implementation in R. New York, USA: Springer; 2015.

[40] Yee TW, Wild CJ. Vector Generalized Additive Models. J Royal Stat Soc - Ser B. 1996;58(3):481–93.

[41] Zeileis A, Grothendieck G. zoo: S3 Infrastructure for Regular and Irregular Time Series. J Stat Softw. 2005;14(6):1–27.

[42] Branson J, Good N, Chen JW, Monge W, Probst C, El Emam K. Evaluating the re-identification risk of a clinical study report anonymized under EMA Policy 0070 and Health Canada Regulations. Trials. 2020;21(1):200. 32070405 10.1186/s13063-020-4120-y PMC7029478

[43] Young RD, Medford A. (World) Supercentenarian Database. 2021.

[44] Anatomisch-Therapeutisch-Chemischen Klassifikation (ATC-Klassifikation) mit definierten Tagesdosen (DDD) der Weltgesundheitsorganisation und den Anpassungen entsprechend der deutschen Versorgungssituation: Bundesinstitut für Medizinprodukte und Arzneimittel (BfArM) im Auftrag des Bundesministeriums für Gesundheit; [Accessed 2024/11/20]. Available from: https://www.bfarm.de/EN/Home/_node.html.

[45] ICD-10-GM Version 2020, Systematisches Verzeichnis, Internationale statistische Klassifikation der Krankheiten und verwandter Gesundheitsprobleme, 10. Revision, Stand: 20. September 2019: Deutsches Institut für Medizinische Dokumentation und Information (DIMDI) im Auftrag des Bundesministeriums für Gesundheit (BMG) unter Beteiligung der Arbeitsgruppe ICD des Kuratoriums für Fragen der Klassifikation im Gesundheitswesen (KKG); 2019 [Accessed 2024/11/20]. Available from: https://www.bfarm.de/EN/Home/_node.html.

[46] ICD-10-GM Version 2021, Systematisches Verzeichnis, Internationale statistische Klassifikation der Krankheiten und verwandter Gesundheitsprobleme, 10. Revision, Stand: 18. September 2020: Bundesinstitut für Arzneimittel und Medizinprodukte (BfArM) im Auftrag des Bundesministeriums für Gesundheit (BMG) unter Beteiligung der Arbeitsgruppe ICD des Kuratoriums für Fragen der Klassifikation im Gesundheitswesen; 2020 [Accessed 2024/11/20]. Available from: https://www.bfarm.de/EN/Home/_node.html.

[47] Quan H, Li B, Couris CM, Fushimi K, Graham P, Hider P, et al. Updating and validating the Charlson comorbidity index and score for risk adjustment in hospital discharge abstracts using data from 6 countries. Am J Epidemiol. 2011;173(6):676–82. 21330339 10.1093/aje/kwq433

[48] Pharmazentralnummer (PZN): Informationsstelle für Arzneispezialitäten (IFA) GmbH; [Accessed 2024/11/20]. Available from: https://www.ifaffm.de/en/home.html.

[49] Balduzzi S, Rucker G, Schwarzer G. How to perform a meta-analysis with R: a practical tutorial. Evid Based Ment Health. 2019;22(4):153–60. 31563865 10.1136/ebmental-2019-300117 PMC10231495

[50] McGrath S, Sohn H, Steele R, Benedetti A. Meta-analysis of the difference of medians. Biom J. 2020;62(1):69–98. 31553488 10.1002/bimj.201900036

[51] McGrath S, Zhao X, Qin ZZ, Steele R, Benedetti A. One-sample aggregate data meta-analysis of medians. Stat Med. 2019;38(6):969–84. 30460713 10.1002/sim.8013

[52] LOINC (Logical Observation Identifiers Names and Codes): Bundesinstitut für Medizinprodukte und Arzneimittel (BfArM); [Accessed 2024/11/20]. Available from: https://www.bfarm.de/EN/Home/_node.html.

[53] McDonald CJ, Huff SM, Suico JG, Hill G, Leavelle D, Aller R, et al. LOINC, a universal standard for identifying laboratory observations: a 5-year update. Clin Chem. 2003;49(4):624–33. 12651816 10.1373/49.4.624

[54] Rosenau L, Behrend P, Wiedekopf J, Gruendner J, Ingenerf J. Uncovering Harmonization Potential in Health Care Data Through Iterative Refinement of Fast Healthcare Interoperability Resources Profiles Based on Retrospective Discrepancy Analysis: Case Study. JMIR Med Inf. 2024;12:e57005. 10.2196/57005 PMC11303887 39042420

[55] Goldstein BA, Navar AM, Pencina MJ, Ioannidis JP. Opportunities and challenges in developing risk prediction models with electronic health records data: a systematic review. J Am Med Inform Assoc. 2017;24(1):198–208. 27189013 10.1093/jamia/ocw042 PMC5201180

[56] Kharrazi H, Wang C, Scharfstein D. Prospective EHR-based clinical trials: the challenge of missing data. J Gen Intern Med. 2014;29(7):976–8. 24839057 10.1007/s11606-014-2883-0 PMC4061350

[57] Kohane IS, Aronow BJ, Avillach P, Beaulieu-Jones BK, Bellazzi R, Bradford RL, et al. What Every Reader Should Know About Studies Using Electronic Health Record Data but May Be Afraid to Ask. J Med Internet Res. 2021;23(3):e22219. 33600347 10.2196/22219 PMC7927948

[58] Rusanov A, Weiskopf NG, Wang S, Weng C. Hidden in plain sight: bias towards sick patients when sampling patients with sufficient electronic health record data for research. BMC Med Inf Decis Mak. 2014;11(14):51. 10.1186/1472-6947-14-51 PMC4062889 24916006

[59] Goldstein BA, Bhavsar NA, Phelan M, Pencina MJ. Controlling for Informed Presence Bias Due to the Number of Health Encounters in an Electronic Health Record. Am J Epidemiol. 2016;184(11):847–55. 27852603 10.1093/aje/kww112 PMC5152663

[60] Miller WG, Tate JR, Barth JH, Jones GR. Harmonization: the sample, the measurement, and the report. Annals Lab Med. 2014;34(3):187–97. 10.3343/alm.2014.34.3.187 PMC3999316 24790905

[61] Kietsiriroje N, Pearson S, Campbell M, Ariens RAS, Ajjan RA. Double Diabetes: A distinct high-risk group? Diabetes, Obesity and Metabolism. 2019;21(12):2609–18. 10.1111/dom.13848 31373146

[62] Brat GA, Weber GM, Gehlenborg N, Avillach P, Palmer NP, Chiovato L, et al. International electronic health record-derived COVID-19 clinical course profiles: the 4CE consortium. NPJ Digit Med. 2020;3:109. 32864472 10.1038/s41746-020-00308-0 PMC7438496

[63] Moal B, Orieux A, Ferte T, Neuraz A, Brat GA, Avillach P, et al. Acute respiratory distress syndrome after SARS-CoV-2 infection on young adult population: International observational federated study based on electronic health records through the 4CE consortium. PLoS ONE. 2023;18(1):e0266985. 36598895 10.1371/journal.pone.0266985 PMC9812312

[64] World Health Organization. International statistical classification of diseases and related health problems, 10th revision, Fifth edition, 2016. 2015 [Accessed 2025/02/26]. Available from: https://iris.who.int/handle/10665/246208.

[65] Regenstrief Institute Inc., UCUM Organization. Unified code for units of measure (UCUM). since 1998.

[66] UCUM-Werteliste zur Kodierung von Einheiten für gesetzliche Anwendungen von elektronischen Dokumenten im Gesundheitswesen in Deutschland: Bundesinstitut für Medizinprodukte und Arzneimittel (BfArM); [Accessed 2024/11/20]. Available from: https://www.bfarm.de/EN/Home/_node.html.

[67] The UCUM-LHC Validator and Converter: National Library of Medicine; [Accessed 2024/11/29]. Available from: https://ucum.nlm.nih.gov/ucum-lhc/demo.html.

[68] MMI Germany GmbH (Vidal MMI). MMI Pharmindex. Langen, Germany.

[69] Medizinformatik-Initiative. Kerndatensatz Modul Medikation 2023 [Accessed 2024/11/20]. Available from: https://www.medizininformatik-initiative.de/Kerndatensatz/Modul_Medikation_Version_2/MIIIGModulMedikation.html.

[70] Prokosch HU, Gebhardt M, Gruendner J, Kleinert P, Buckow K, Rosenau L, Semler SC. Towards a National Portal for Medical Research Data (FDPG): Vision, Status, and Lessons Learned. Stud Health Technol Inform. 2023;302:307–11. 37203668 10.3233/SHTI230124

[71] Lee KJ, Tilling KM, Cornish RP, Little RJA, Bell ML, Goetghebeur E, et al. Framework for the treatment and reporting of missing data in observational studies: The Treatment And Reporting of Missing data in Observational Studies framework. J Clin Epidemiol. 2021;134:79–88. 33539930 10.1016/j.jclinepi.2021.01.008 PMC8168830

[72] Gaye A, Marcon Y, Isaeva J, LaFlamme P, Turner A, Jones EM, et al. DataSHIELD: taking the analysis to the data, not the data to the analysis. Int J Epidemiol. 2014;43(6):1929–44. 25261970 10.1093/ije/dyu188 PMC4276062

[73] Beyan O, Choudhury A, van Soest J, Kohlbacher O, Zimmermann L, Stenzhorn H, et al. Distributed analytics on sensitive medical data: The Personal Health Train. Data Intell. 2020;2(1–2):96–107.

[74] Hripcsak G, Duke JD, Shah NH, Reich CG, Huser V, Schuemie MJ, et al. Observational Health Data Sciences and Informatics (OHDSI): opportunities for observational researchers. Stud Health Technol Inform. 2015;216:574-8. PMC4815923 26262116

[75] Loeffler M, Maas R, Neumann D, Scherag A. INTERPOLAR – prospektive, interventionelle Studien im Rahmen der Medizininformatik-Initiative zur Verbesserung der Arzneimitteltherapiesicherheit in der Krankenversorgung. Bundesgesundheitsblatt Gesundheitsforschung Gesundheitsschutz. 2024;67(6):676–84. 38750238 10.1007/s00103-024-03890-w PMC11166858

