# Supplementary material for: A distributed analysis approach for pharmacovigilance data from electronic medical records in German university hospitals: the POLAR_MI ETL Pipeline
Source: BMC Med Inform Decis Mak. 2026 Jun 15;26:220. doi: 10.1186/s12911-026-03550-w (PMC13270667; doi:10.1186/s12911-026-03550-w)
Supplement: Supplementary file 2 — Supplementary Material 2: Additional File 2: Supplementary Tables (PDF) [file 12911_2026_3550_MOESM2_ESM.pdf]

Additional File 2: Supplementary Tables

A distributed analysis approach for pharmacovigilance data from electronic medical records in German university hospitals: the POLAR_MI ETL Pipeline

Miriam Kesselmeier[1,#], Torsten Thalheim[2,3,4], Florian Schmidt[3], Thomas Peschel[3], Julia Palm[1], Alexander Strübing[3], André Medek[5], Jens Przybilla[3,6], Anna Maria Wermund[7], Renke Maas[8], Steffen Härterich[9], Louisa Redeker[10], Martin Federbusch[11], Daniel Steinbach[11], Jan Gewehr[12], Marcus Wurlitzer[12], Andrea Riedel[13,14], Frank Meineke[3], Daniel Neumann[3], André Scherag[1,*] and Markus Loeffler[3,*] on behalf of POLAR_MI[+]

[1] Institute of Medical Statistics, Computer and Data Sciences (IMSID), Jena University Hospital – Friedrich Schiller University Jena, Jena, Germany

[2] Interdisciplinary Centre for Bioinformatics, Leipzig University, Leipzig, Germany

[3] Institute for Medical Informatics, Statistics and Epidemiology (IMISE), Leipzig University, Leipzig, Germany

[4] Deutsches Biomasseforschungszentrum gGmbH, Torgauer Str. 116, 04347 Leipzig, Germany

[5] Medical & Scientific Technology Development & Coordination (MWTek), University Hospital Bonn, Bonn, Germany

[6] Clinical Trial Centre Leipzig (ZKS), Leipzig University, Leipzig, Germany

[7] Department of Clinical Pharmacy, Institute of Pharmacy, University of Bonn, Bonn, Germany

[8] Institute of Experimental and Clinical Pharmacology and Toxicology, Friedrich-Alexander-Universität Erlangen-Nürnberg, Erlangen, Germany

[9] Hospital Pharmacy, University Medical Center Hamburg-Eppendorf, Hamburg, Germany

[10] Department of Clinical Pharmacology, School of Medicine, Faculty of Health, Witten/Herdecke University, Witten, Germany

[11] Institute for Laboratory Medicine, Clinical Chemistry and Molecular Diagnostics, University Medical Center Leipzig, Leipzig, Germany

[12] Business Division for Information Technology, University Medical Center Hamburg-Eppendorf, Hamburg, Germany

[13] Erlangen University Hospital, Medical Center for Information and Communication Technology, Erlangen, Germany

[14] Friedrich-Alexander-Universität Erlangen-Nürnberg, Medical Informatics, Erlangen, Germany

[#] Corresponding author
Email: miriam.kesselmeier@med.uni-jena.de

[*] Equal contribution

[+] The membership list of POLAR_MI is provided in Additional File 1.

List of tables

Supplementary Table 1. R packages and their version used within the Docker for local analysis 3

Supplementary Table 2. Reasons for technical exclusions 4

Supplementary Table 3. Additional definition details required for proving inclusion criteria as well as for the population description 6

Supplementary Table 4. Observed time stamp integration for medications, laboratory values and diagnoses 7

Supplementary Table 5. Definition of medication-related proxy variables 8

Supplementary Table 6. Integration of laboratory values 9

Supplementary Table 7. Meta-analysed description of the POLAR_MI population and of the modified POLAR_MI population across ten centres. 10

Supplementary Table 8. Meta-analysed description of availability of medication resources and medication systems in the modified POLAR_MI population 13

Supplementary Table 9. Meta-analysed description of the modified POLAR_MI population reduced by encounters without 7-character ATC code 14

Supplementary Table 1. R packages and their version used within the Docker for local analysis.

Local analysis part	R package		
	Name	Version	Reference
Data retrieval	data.table	1.14.8	(1)
	dplyr	1.1.2	(2)
	fhircrackr	2.1.1	(3)
	ggplot2	3.4.3	(4)
	readxl	1.4.3	(5)
	xml2	1.3.5	(6)
Statistical analysis	bestglm	0.37.3	(7)
	data.table	1.14.8	(1)
	DescTools	0.99.49	(8)
	dplyr	1.1.2	(2)
	fhircrackr	2.1.1	(3)
	ggplot2	3.4.3	(4)
	gtools	3.9.4	(9)
	leaps	3.1	(10)
	lmtest	0.9-40	(11)
	lubridate	1.9.2	(12)
	mice	3.16.0	(13)
	oddsratio	2.0.1	(14)
	randomForest	4.7-1.1	(15)
	readxl	1.4.3	(5)
	regclass	1.6	(16)
	robustbase	0.99-0	(17)
	ROCit	2.1.1	(18)
	rpart	4.1.19	(19)
	VGAM	1.1-8	(20, 21)
	xml2	1.3.5	(6)
	zoo	1.8-12	(22)

Supplementary Table 2. Reasons for technical exclusions.

Issue	Reason for exclusion
Exclusion of encounters	
Encounter with invalid or not unique patient reference	Mandatory information
Encounter with missing/invalid hospital admission and/or discharge date	Mandatory information
Encounter with an invalid resource	Introduction of bias possible if parts of the resources cannot be used
Encounters of a patient with time overlap in their hospital stays	A person cannot exist (and be included in the analysis) several times in parallel. The union of these encounters bears risks, so that we decided to exclude these encounters.<br>Note: A possible source for these overlapping stays are in-house incidents, if a ward transfer of a patient, for example, caused multiple documentation of a single hospital stay. In these circumstances, the CDS definition of an encounter allows to combine several encounters, defined by in-house regulations, to one hospital stay.
Encounter with a start date after its end date	This is a documentation error, but there is no possible workaround.
Encounter whose associated patient has a default birth date or was born before 01/01/1910	Default values can belong to minors that are not allowed to be included in the POLAR_MI analyses. The observed default values induced the introduction of the additional constraint on a patient's birthdate.<br>Note: This approach excluded patients with spelling errors together with a neglectable number of actual supercentenarians. As a latent re-identification risk is present even in anonymised clinical trials (23), the application of a strict lower bound prevented discussion on potential re-identification issues, as supercentenarian Germans have been validated a few times only. Till 2019, the "International Database of Longevity" reported 17 people with a validated age of at least 110 years in Germany for all times (24).
All encounters of a patient, for whom at least one encounter must be excluded	The exclusion of only some encounters would introduce a bias that might be even more difficult to handle than the bias introduced by the exclusion of any encounter.
Resources of an encounter/patient	
Resources of a patient, if there is no unique link between resource and encounter (e.g., no encounter reference, no time overlap, no valid time stamp for assessment of time overlap, resource time stamp within several hospital stays of a patient)	Random allocation would introduce a bias.

Resources of a patient if they can be mapped to multiple encounters	Mandatory cardinality is 1:1 for resource:encounter.
Resource without unique time stamp, i.e., a time point and a time period are specified together	Ambiguous definition, a period comprises multiple, recurrent time points and not a single event.
Resource with a time order violation, i.e., end time a priori to start time	This is a contradiction.
Observation with a LOINC code mismatch	This only applies, if laboratory values need to be downloaded based on patient references and resources must be discarded.
MedicationAdministration and MedicationStatement with invalid medication reference	Information is not usable.
Further reasons	
Analysis-specific reasons	E.g., missing information to check the inclusion criteria

Abbreviations: CDS, core data set; LOINC, Logical Observation Identifiers Names and Codes.

Supplementary Table 3. Additional definition details required for proving inclusion criteria as well as for the population description.

Item	Definition
Inclusion criteria	
Inpatient hospital stay	Encounters were considered as inpatient encounters if they had no so-called “partOf” relationship to other encounters (and therefore did not represent a smaller administrative unit than the hospital stay, which is another type of encounter profile in the CDS) and if they contained one of the following character strings in the XML path "class/code", regardless of upper or lower case: 'station', 'IMP', 'inpatient', 'emer', 'ACUTE' or 'NONAC'. Some of the entries ('IMP', 'ACUTE', 'NONAC' and 'emer') originated from the code system (https://terminology.hl7.org/ValueSet-v3-ActEncounterCode.html) and are intended precisely for coding inpatient stays. Other codes ('station', 'inpatient') were added by agreement with the DIC because this information was not stored in accordance with the standard at some DIC.
Population description	
Medications	• CDS resources: Medication and (MedicationAdministration or MedicationStatement) [MedicationAdministration and MedicationStatement contains the administration/prescription with a reference to the medication in the CDS resource Medication]<br>• In POLAR_MI: provided as 7-character ATC codes
Diagnoses	• CDS resource: Condition<br>• In POLAR_MI: according to ICD-10-GM

Abbreviations: ATC, Anatomical Therapeutic Chemical according to the World Health Organisation; CDS, core data set; DIC, data integration centre; ICD-10-GM, German Modification of the International Statistical Classification of Diseases and Related Health Problems (10th Revision).

Supplementary Table 4. Observed time stamp integration for medications, laboratory values and diagnoses.

Item	Issue
Medications	The meaning of a specific time point was not unique (e.g., time of prescription, time of administration, time of intake, synthetic time point or time interval). Dosing schemes (e.g. “three times a day”) were also often provided instead of specific time points. In some cases, these dosing schemes were translated from categories (“morning”, “noon”, “afternoon”) to assumed times of the day (e.g., 6:00 am, 12:00 pm and 8:00 pm).
Laboratory values	Time stamps for sampling, ordering lab service, sample receipt by laboratory, laboratory analysis and result transmission by laboratory were defined in the CDS specification to enable data integration at a DIC according to their local hospital and laboratory setting. However, at some DIC, all these time stamps were filled identically even across different samples of an encounter. This was critical if those samples provided contrary statements.
Diagnoses	Data according to §21 of the German Hospital Reimbursement Act (“Krankenhausentgeltgesetz”) was used in some DIC. This kind of data has no time stamp usable for analysis, as it is used for reimbursement purposes and often created during coding up to 28 days after the patient’s hospital discharge. Here, our algorithm-based identification of reliable time stamps could not distinguish between retrospectively documented diagnoses and live documentation.

Abbreviation: CDS, core data set; DIC, data integration centre.

Supplementary Table 5. Definition of medication-related proxy variables.

Proxy variable	Definition
Documented medication	At least one of the FHIR resources MedicationStatement and MedicationAdministration were filled<br>Note: Although the FHIR resources MedicationStatement and MedicationAdministration with their semantic implications (record versus consumption) were described in the CDS specifications, these two resources were confused by some DIC. Furthermore, some DIC provided one of these two medication resources only. Hence, we could not distinguish MedicationAdministration and MedicationStatement and included all available information in the analyses. Of note, the medication resources MedicationRequest and MedicationList, that may have the potential to overcome these issues, were not ready to use during POLAR_MI.
Ambulant medication	Documented medication with a time stamp at the day of hospital admission or earlier
Discharge recommendation	Documented medication with a time stamp on the day of hospital discharge or later

Abbreviations: CDS, core data set; DIC, data integration centre; FHIR, Fast Health Interoperability Resources.

Supplementary Table 6. Integration of laboratory values.

Laboratory value	Integration
Overall	
Renal measurements	Units were ignored, as the magnitude of the renal measurement value was independent from the permitted units.
Preference of certain LOINC codes	For some analyses, we defined LOINC code rankings, specifying a preference of certain LOINC codes to be evaluated primarily. This acknowledgement introduced heterogeneity but enabled to include more DIC into the analysis. Without ranking, all defined LOINC codes were treated equally.
Categorised laboratory values	For some analyses, only categorised laboratory values in relation to the reference range (e.g., normal range / increased value / decreased value) were required. In this case, the related category thresholds were provided for all expected units within the locally executed part of the POLAR_MI ETL Pipeline.
Contradictory resources within one encounter	
Several, different eGFR values with identical time stamps	In case only the information is required, whether the eGFR is below a certain threshold (e.g., 30 ml/min), the most severe eGFR value might be used.
Several serum albumin values, which were categorised to different categories (e. g., decreased, normal and increased), with identical time stamps	Two-fold: (i) If a decreased and a normal value or an increased and a normal value are observed together, the aberrant value might be used. (ii) A decreased and an increased value together is a contradiction and, consequently, the value is set to unknown.
Rare events	
Specific laboratory category: increased serum albumin	This category was observed in few DIC only. Encounters with increased serum albumin were removed from regression modelling, but assessed in the related population description. Otherwise, regression models estimated at different DIC differ by the number of regression coefficients, which hinder the subsequent meta-analysis.

Abbreviations: DIC, data integration centre; eGFR, estimated glomerular filtration rate; LOINC, Logical Observation Identifiers Names and Codes.

Supplementary Table 7. Meta-analysed description of the POLAR_MI population and of the modified POLAR_MI population across ten centres.

Characteristic	POLAR_MI population		Modified POLAR_MI population	
	N	Distribution (95% CI)	N	Distribution (95% CI)
Total number of encounters	788,127		713,018	
Total number of patients	501,613		489,267	
Age, in years	788,127			
Overall		60.10 (58.13, 62.07)	713,018	60.00 (58.02, 61.98)
Encounters aged 18-40 years		22.48% (19.32%, 25.99%)	713,018	22.81% (19.84%, 26.09%)
Encounters aged 41-64 years		34.97% (33.57%, 36.40%)	713,018	34.91% (33.60%, 36.23%)
Encounters aged 65 years and older		41.99% (38.99%, 45.04%)	713,018	41.78% (38.95%, 44.67%)
Sex				
Male sex	788,127	50.54% (48.82%, 52.27%)	713,018	50.23% (48.59%, 51.86%)
Median age, in years	395,655	62.60 (61.05, 64.15)	355,386	62.40 (60.96, 63.84)
Encounters aged 18-40 years	180,100	36.89% (33.37%, 40.54%)	167,411	36.66% (33.06%, 40.41%)
Encounters aged 41-64 years	277,949	54.10% (52.61%, 55.58%)	250,376	54.15% (52.73%, 55.57%)
Encounters aged 65 years and older	330,078	55.19% (53.80%, 56.57%)	295,231	54.64% (53.42%, 55.86%)
Female sex	788,127	49.45% (47.73%, 51.18%)	713,018	49.77% (48.13%, 51.40%)
Median age, in years	392,435	56.60 (53.79, 59.41)	357,598	56.70 (53.94, 59.46)
Encounters aged 18-40 years	180,100	63.11% (59.45%, 66.62%)	167,411	63.33% (59.59%, 66.93%)
Encounters aged 41-64 years	277,949	45.90% (44.42%, 47.38%)	250,376	45.84% (44.43%, 47.26%)
Encounters aged 65 years and older	330,078	44.81% (43.43%, 46.20%)	295,231	45.36% (44.14%, 46.58%)
Sex divers or missing sex information	788,127	0.00% (0.00%, 0.01%)	713,018	0.00% (0.00%, 0.01%)
Median age, in years	37	not estimable	34	not estimable
Encounters aged 18-40 years	180,100	0.01% (0.00%, 0.01%)	167,411	0.01% (0.00%, 0.01%)
Encounters aged 41-64 years	277,949	0.00% (0.00%, 0.01%)	250,376	0.00% (0.00%, 0.01%)
Encounters aged 65 years and older	330,078	0.00% (0.00%, 0.01%)	295,231	0.00% (0.00%, 0.01%)
Length of stay, in days				
Overall	788,127	3.00 (2.17, 3.83)	713,018	3.10 (2.25, 3.95)
Encounters aged 18-40 years	180,100	2.70 (2.04, 3.36)	167,411	2.70 (2.04, 3.36)
Encounters aged 41-64 years	277,949	2.80 (2.10, 3.50)	250,376	2.80 (2.10, 3.50)
Encounters aged 65 years and older	330,078	3.60 (2.82, 4.38)	295,231	3.50 (2.77, 4.23)
Encounters with male sex	395,655	3.10 (2.42, 3.78)	355,386	3.20 (2.50, 3.90)
Encounters with female sex	392,435	2.90 (2.10, 3.70)	357,598	3.00 (2.17, 3.83)

Encounters with sex divers or missing sex information	37	not estimable	34	not estimable
Proportion of encounters with diagnosis information available				
Overall	788,127	98.61% (90.03%, 99.82%)	713,018	99.27% (94.29%, 99.91%)
Encounters aged 18-40 years	180,100	97.82% (87.34%, 99.66%)	167,411	98.67% (91.52%, 99.80%)
Encounters aged 41-64 years	277,949	98.49% (90.71%, 99.77%)	250,376	99.20% (94.54%, 99.89%)
Encounters aged 65 years and older	330,078	99.41% (95.67%, 99.92%)	295,231	99.74% (97.96%, 99.97%)
Encounters with male sex	395,655	98.78% (92.05%, 99.82%)	355,386	99.33% (95.47%, 99.91%)
Encounters with female sex	392,435	98.37% (89.35%, 99.77%)	357,598	99.18% (93.77%, 99.90%)
Encounters with sex divers or missing sex information	37	not estimable	34	not estimable
Number of different diagnoses per encounter*1				
Overall	705,490	6.80 (6.16, 7.44)	657,054	6.80 (6.16, 7.44)
Encounters aged 18-40 years	157,659	4.90 (4.36, 5.44)	149,041	4.90 (4.36, 5.44)
Encounters aged 41-64 years	246,753	6.10 (5.56, 6.64)	229,262	6.10 (5.56, 6.64)
Encounters aged 65 years and older	301,078	8.70 (8.05, 9.36)	278,751	8.80 (8.04, 9.56)
Encounters with male sex	357,454	7.00 (6.35, 7.65)	331,277	7.00 (6.35, 7.65)
Encounters with female sex	348,008	6.50 (5.90, 7.10)	325,749	6.50 (5.90, 7.10)
Encounters with sex divers or missing sex information	28	not estimable	28	not estimable
Charlson comorbidity index with age, in points*1				
Overall	705,490	2.50 (2.17, 2.83)	657,054	2.30 (2.00, 2.60)
Encounters aged 18-40 years	157,659	0.00 (0.00, 0.00)	149,041	0.00 (0.00, 0.00)
Encounters aged 41-64 years	246,753	1.70 (1.40, 2.00)	229,262	1.70 (1.40, 2.00)
Encounters aged 65 years and older	301,078	4.10 (3.90, 4.30)	278,751	4.10 (3.90, 4.30)
Encounters with male sex	357,454	2.90 (2.70, 3.10)	331,277	2.90 (2.70, 3.10)
Encounters with female sex	348,008	2.20 (1.81, 2.59)	325,749	2.10 (1.75, 2.45)
Encounters with sex divers or missing sex information	28	not estimable	28	not estimable
Proportion of encounters with 7-character ATC codes available				
Overall	788,127	63.71% (31.91%, 86.81%)	713,018	65.84% (33.72%, 87.96%)
Encounters aged 18-40 years	180,100	50.23% (21.42%, 78.89%)	167,411	51.37% (22.01%, 79.82%)
Encounters aged 41-64 years	277,949	64.36% (31.37%, 87.70%)	250,376	66.30% (33.17%, 88.64%)
Encounters aged 65 years and older	330,078	71.81% (38.35%, 91.25%)	295,231	74.28% (41.13%, 92.27%)
Encounters with male sex	395,655	66.55% (32.98%, 88.94%)	355,386	68.84% (34.93%, 90.09%)
Encounters with female sex	392,435	61.28% (30.61%, 85.03%)	357,598	63.27% (32.23%, 86.18%)
Encounters with sex divers or missing sex information	37	not estimable	34	not estimable

Number of different 7-character ATC codes per encounter[*2]				
Overall	397,536	6.60 (5.05, 8.15)	365,322	6.60 (5.05, 8.15)
Encounters aged 18-40 years	76,834	4.10 (2.54, 5.66)	71,832	4.10 (2.54, 5.65)
Encounters aged 41-64 years	138,972	6.10 (4.49, 7.71)	127,364	6.10 (4.49, 7.71)
Encounters aged 65 years and older	181,730	8.30 (6.75, 9.85)	166,126	8.30 (6.75, 9.85)
Encounters with male sex	206,373	7.00 (5.43, 8.57)	188,012	7.00 (5.43, 8.57)
Encounters with female sex	191,143	6.20 (4.58, 7.82)	177,292	6.10 (4.46, 7.74)
Encounters with sex divers or missing sex information	20	not estimable	18	not estimable

Results are provided overall as well as stratified by sex and age categories. Proportion and median, respectively, with 95% confidence interval (CI) are provided. Additionally, the number of encounters/patients (N) building the underlying sample for the respective characteristic (in the given strata) is provided. The number of encounters with missing information of the respective characteristic is provided as a footnote, if necessary. The modified POLAR_MI population originated from the POLAR_MI population by excluding encounters fulfilling technical exclusion reasons (see Figure 2). In our analysis, an encounter is equivalent to a case, i.e., a hospital stay of a patient.

[*1] POLAR_MI population: Information missing for 82,637 encounters (stratified by category: 22,441 encounters aged 18-40 years, 31,196 encounters aged 41-64 years, 29,000 encounters aged 65 years and older, 38,201 encounters with male sex, 44,427 encounters with female sex, 9 encounters with sex divers or missing sex information). Modified POLAR_MI population: Information missing for 55,964 encounters (stratified by category: 18,370 encounters aged 18-40 years, 21,114 encounters aged 41-64 years, 16,480 encounters aged 65 years and older, 24,109 encounters with male sex, 31,849 encounters with female sex, 6 encounters with sex divers or missing sex information).

[*2] POLAR_MI population: Information missing for 390,591 encounters (stratified by category: 103,266 encounters aged 18-40 years, 138,977 encounters aged 41-64 years, 148,348 encounters aged 65 years and older, 189,282 encounters with male sex, 201,292 encounters with female sex, 17 encounters with sex divers or missing sex information). Modified POLAR_MI population: Information missing for 347,696 encounters (stratified by category: 95,579 encounters aged 18-40 years, 123,012 encounters aged 41-64 years, 129,105 encounters aged 65 years and older, 167,374 encounters with male sex, 180,306 encounters with female sex, 16 encounters with sex divers or missing sex information).

Supplementary Table 8. Meta-analysed description of availability of medication resources and medication systems in the modified POLAR_MI population.

Availability of …			Proportion (95% CI), in %
… medication resources	… ATC code	… PZN	
All encounters (N = 713,018)			
no	-	-	32.50 (10.23, 67.05)
yes	-	-	67.50 (32.91, 89.79)
Encounters (N = 366,225) with available medication resources			
yes	-	no	0.04 (0.00, 0.38)
yes	-	yes, any	99.96 (99.62, 100.00)
yes	-	yes, 8-digit	99.87 (98.79, 99.99)
yes	no	-	0.01 (0.00, 0.05)
yes	no	no	0.00 (0.00, 0.01)
yes	no	yes, any	0.01 (0.00, 0.04)
yes	no	yes, 8-digit	0.01 (0.00, 0.04)
yes	yes, any	-	99.99 (99.95, 100.00)
yes	yes, any	no	0.02 (0.00, 0.39)
yes	yes, any	yes, any	99.88 (99.50, 99.97)
yes	yes, any	yes, 8-digit	99.72 (98.58, 99.95)
yes	yes, 7-character	-	99.95 (99.86, 99.98)
yes	yes, 7-character	no	0.02 (0.00, 0.37)
yes	yes, 7-character	yes, any	99.84 (99.33, 99.96)
yes	yes, 7-character	yes, 8-digit	99.63 (98.21, 99.92)

Proportions with 95% confidence interval (CI) are provided. Furthermore, the number of encounters (N) building the underlying sample for proportion estimation are provided. Note, despite the fact that ATC codes with less than seven characters are valid, they disallow an analysis with specific medications. For PZN, the former number exhibited seven digits, which cannot be used when relying on the current version with eight digits. Usually, an encounter is equivalent to a case, i.e. a hospital stay of a patient. Abbreviations: ATC, anatomical therapeutic chemical; PZN, Pharmazentralnummer (pharmaceutical central number).

Supplementary Table 9. Meta-analysed description of the modified POLAR_MI population reduced by encounters without documented 7-character ATC code.

Characteristic	N	Distribution (95% CI)
Total number of encounters	365,344	
Total number of patients	270,384	
Age, in years	365,344	
Overall		62.90 (61.09, 64.71)
Encounters aged 18-40 years		17.20% (14.09%, 20.84%)
Encounters aged 41-64 years		35.02% (33.29%, 36.79%)
Encounters aged 65 years and older		47.00% (43.66%, 50.37%)
Sex		
Male sex	365,344	51.71% (49.51%, 53.90%)
Median age, in years	188,012	64.00 (62.45, 65.54)
Encounters aged 18-40 years	71,854	39.47% (34.25%, 44.94%)
Encounters aged 41-64 years	127,364	55.29% (53.54%, 57.02%)
Encounters aged 65 years and older	166,126	54.20% (52.20%, 56.18%)
Female sex	365,344	48.28% (46.09%, 50.48%)
Median age, in years	177,314	60.99 (58.22, 63.76)
Encounters aged 18-40 years	71,854	60.53% (55.06%, 65.75%)
Encounters aged 41-64 years	127,364	44.70% (42.97%, 46.45%)
Encounters aged 65 years and older	166,126	45.80% (43.82%, 47.80%)
Sex divers or missing sex information	365,344	0.00% (0.00%, 0.01%)
Median age, in years	18	not estimable
Encounters aged 18-40 years	71,854	0.00% (0.00%, 0.03%)
Encounters aged 41-64 years	127,364	0.00% (0.00%, 0.03%)
Encounters aged 65 years and older	166,126	0.00% (0.00%, 10.76%)
Length of stay, in days		
Overall	365,344	4.00 (3.59, 4.41)
Encounters aged 18-40 years	71,854	3.28 (2.99, 3.57)
Encounters aged 41-64 years	127,364	3.99 (3.58, 4.40)
Encounters aged 65 years and older	166,126	4.60 (4.00, 5.20)
Encounters with male sex	188,012	4.00 (3.58, 4.41)
Encounters with female sex	177,314	3.99 (3.58, 4.40)
Encounters with sex divers or missing sex information	18	not estimable
Proportion of encounters with diagnosis		
Overall	365,344	99.93% (98.80%, 100.00%)
Encounters aged 18-40 years	71,854	99.84% (98.41%, 99.99%)
Encounters aged 41-64 years	127,364	99.93% (98.87%, 100.00%)
Encounters aged 65 years and older	166,126	99.97% (99.01%, 100.00%)
Encounters with male sex	188,012	99.96% (98.82%, 100.00%)
Encounters with female sex	177,314	99.91% (98.83%, 99.99%)
Encounters with sex divers or missing sex information	18	not estimable
Number of different diagnoses per encounter[*1]		
Overall	355,558	7.90 (7.16, 8.64)
Encounters aged 18-40 years	70,220	5.81 (5.11, 6.51)
Encounters aged 41-64 years	123,586	7.20 (6.56, 7.84)
Encounters aged 65 years and older	161,752	9.30 (8.52, 10.08)
Encounters with male sex	182,531	8.00 (7.23, 8.77)
Encounters with female sex	173,009	7.70 (6.98, 8.42)
Encounters with sex divers or missing sex information	18	not estimable
Charlson comorbidity index with age, in points[*1]		

Overall	355,558	2.80 (2.41, 3.19)
Encounters aged 18-40 years	70,220	0.00 (0.00, 0.00)
Encounters aged 41-64 years	123,586	1.80 (1.54, 2.06)
Encounters aged 65 years and older	161,752	4.10 (3.90, 4.30)
Encounters with male sex	182,531	3.20 (2.94, 3.46)
Encounters with female sex	173,009	2.60 (2.08, 3.12)
Encounters with sex divers or missing sex information	18	not estimable
Proportion of encounters with 7-character ATC classification		
Overall	365,322	100.00% (0.00%, 100.00%)
Encounters aged 18-40 years	71,832	100.00% (0.00%, 100.00%)
Encounters aged 41-64 years	127,364	100.00% (0.00%, 100.00%)
Encounters aged 65 years and older	166,126	100.00% (0.00%, 100.00%)
Encounters with male sex	188,012	100.00% (0.00%, 100.00%)
Encounters with female sex	177,292	100.00% (0.00%, 100.00%)
Encounters with sex divers or missing sex information	18	not estimable
Number of different 7-character ATC classifications per encounter		
Overall	365,322	6.60 (5.05, 8.15)
Encounters aged 18-40 years	71,832	4.10 (2.54, 5.65)
Encounters aged 41-64 years	127,364	6.10 (4.49, 7.71)
Encounters aged 65 years and older	166,126	8.30 (6.75, 9.85)
Encounters with male sex	188,012	7.00 (5.43, 8.57)
Encounters with female sex	177,292	6.10 (4.46, 7.74)
Encounters with sex divers or missing sex information	18	not estimable

Results are provided overall as well as stratified by sex and age categories. Proportion and median, respectively, with 95% confidence interval (CI) are provided. Additionally, the number of encounters/patients (N) building the underlying sample for the respective characteristic (in the given strata) is provided. The number of encounters with missing information of the respective characteristic is provided as a footnote, if necessary. In our analysis, an encounter is equivalent to a case, i.e., a hospital stay of a patient.

*1 Information missing for 9,786 encounters (stratified by category: 1,634 encounters aged 18-40 years, 3,778 encounters aged 41-64 years, 4,374 encounters aged 65 years and older, 5,481 encounters with male sex, 4,305 encounters with female sex, 0 encounters with sex divers or missing sex information).

References

1. Barrett T, Dowle M, Srinivasan A, Gorecki J, Chirico M, Hocking T. data.table: Extension of `data.frame` [R package]. Available from: https://CRAN.R-project.org/package=data.table.
2. Wickham H, François R, Henry L, Müller K, Vaughan D. dplyr: A Grammar of Data Manipulation [R package]. Available from: https://CRAN.R-project.org/package=dplyr.
3. Palm J, Meineke FA, Przybilla J, Peschel T. "fhircrackr": An R Package Unlocking Fast Healthcare Interoperability Resources for Statistical Analysis. Applied Clinical Informatics. 2023;14(1):54-64.
4. Wickham H. ggplot2: Elegant Graphics for Data Analysis. New York, USA: Springer; 2016.
5. Wickham H, Bryan J. readxl: Read Excel Files [R package]. Available from: https://CRAN.R-project.org/package=readxl.
6. Wickham H, Hester J, Ooms J. xml2: Parse XML [R package]. Available from: https://CRAN.R-project.org/package=xml2.
7. McLeod A, Xu C, Lai Y. bestglm: Best Subset GLM and Regression Utilities [R package]. Available from: https://CRAN.R-project.org/package=bestglm.
8. Signorell A. DescTools: Tools for Descriptive Statistics [R package]. Available from: https://CRAN.R-project.org/package=DescTools.
9. Warnes G, Bolker B, Lumley T, Magnusson A, Venables B, Ryodan G, et al. gtools: Various R Programming Tools [R package]. Available from: https://CRAN.R-project.org/package=gtools.
10. Lumley T, Miller A. leaps: Regression Subset Selection [R package]. Available from: https://CRAN.R-project.org/package=leaps.
11. Zeileis A, Hothorn T. Diagnostic Checking in Regression Relationships. R News. 2002;2(3):7-10.
12. Grolemund G, Wickham H. Dates and Times Made Easy with lubridate. Journal of Statistical Software. 2011;40(3):1-25.
13. van Buuren S, Groothuis-Oudshoorn K. mice: Multivariate Imputation by Chained Equations in R. Journal of Statistical Software. 2011;45(3):1-67.
14. Schratz P. R package 'oddsratio': Odds ratio calculation for GAM(M)s & GLM(M)s [R package]. Available from: https://CRAN.R-project.org/package=oddsratio.
15. Liaw A, Wiener M. Classification and Regression by randomForest. R News. 2002;2(3):18-22.
16. Petrie A. regclass: Tools for an Introductory Class in Regression and Modeling [R package]. Available from: https://CRAN.R-project.org/package=regclass.
17. Maechler M, Rousseeuw P, Croux C, Todorov V, Ruckstuhl A, Salibian-Barrera M, et al. robustbase: Basic Robust Statistics [R package]. Available from: http://CRAN.R-project.org/package=robustbase.
18. Khan MRA, Brandenburger T. ROCit: Performance Assessment of Binary Classifier with Visualization [R package]. Available from: https://CRAN.R-project.org/package=ROCit.
19. Therneau T, Atkinson B. rpart: Recursive Partitioning and Regression Trees [R package]. Available from: https://CRAN.R-project.org/package=rpart.
20. Yee TW. Vector Generalized Linear and Additive Models: With an Implementation in R. New York, USA: Springer; 2015.
21. Yee TW, Wild CJ. Vector Generalized Additive Models. Journal of Royal Statistical Society - Series B. 1996;58(3):481-93.
22. Zeileis A, Grothendieck G. zoo: S3 Infrastructure for Regular and Irregular Time Series. Journal of Statistical Software. 2005;14(6):1-27.
23. Branson J, Good N, Chen JW, Monge W, Probst C, El Emam K. Evaluating the re-identification risk of a clinical study report anonymized under EMA Policy 0070 and Health Canada Regulations. Trials. 2020;21(1):200.
24. Young RD, Medford A. (World) Supercentenarian Database. 2021.
