# Supplementary material for: A distributed analysis approach for pharmacovigilance data from electronic medical records in German university hospitals: the POLAR_MI ETL Pipeline
Source: BMC Med Inform Decis Mak. 2026 Jun 15;26:220. doi: 10.1186/s12911-026-03550-w (PMC13270667; doi:10.1186/s12911-026-03550-w)
Supplement: Supplementary file 3 — Supplementary Material 3: Additional File 3: Supplementary Figures (PDF) [file 12911_2026_3550_MOESM3_ESM.pdf]

Additional File 3: Supplementary Figures

A distributed analysis approach for pharmacovigilance data from electronic medical records in German university hospitals: the POLAR_MI ETL Pipeline

Miriam Kesselmeier[1,#], Torsten Thalheim[2,3,4], Florian Schmidt[3], Thomas Peschel[3], Julia Palm[1], Alexander Strübing[3], André Medek[5], Jens Przybilla[3,6], Anna Maria Wermund[7], Renke Maas[8], Steffen Härterich[9], Louisa Redeker[10], Martin Federbusch[11], Daniel Steinbach[11], Jan Gewehr[12], Marcus Wurlitzer[12], Andrea Riedel[13,14], Frank Meineke[3], Daniel Neumann[3], André Scherag[1,*] and Markus Loeffler[3,*] on behalf of POLAR_MI[+]

[1] Institute of Medical Statistics, Computer and Data Sciences (IMSID), Jena University Hospital – Friedrich Schiller University Jena, Jena, Germany

[2] Interdisciplinary Centre for Bioinformatics, Leipzig University, Leipzig, Germany

[3] Institute for Medical Informatics, Statistics and Epidemiology (IMISE), Leipzig University, Leipzig, Germany

[4] Deutsches Biomasseforschungszentrum gGmbH, Torgauer Str. 116, 04347 Leipzig, Germany

[5] Medical & Scientific Technology Development & Coordination (MWTek), University Hospital Bonn, Bonn, Germany

[6] Clinical Trial Centre Leipzig (ZKS), Leipzig University, Leipzig, Germany

[7] Department of Clinical Pharmacy, Institute of Pharmacy, University of Bonn, Bonn, Germany

[8] Institute of Experimental and Clinical Pharmacology and Toxicology, Friedrich-Alexander-Universität Erlangen-Nürnberg, Erlangen, Germany

[9] Hospital Pharmacy, University Medical Center Hamburg-Eppendorf, Hamburg, Germany

[10] Department of Clinical Pharmacology, School of Medicine, Faculty of Health, Witten/Herdecke University, Witten, Germany

[11] Institute for Laboratory Medicine, Clinical Chemistry and Molecular Diagnostics, University Medical Center Leipzig, Leipzig, Germany

[12] Business Division for Information Technology, University Medical Center Hamburg-Eppendorf, Hamburg, Germany

[13] Erlangen University Hospital, Medical Center for Information and Communication Technology, Erlangen, Germany

[14] Friedrich-Alexander-Universität Erlangen-Nürnberg, Medical Informatics, Erlangen, Germany

[#] Corresponding author
Email: miriam.kesselmeier@med.uni-jena.de

[*] Equal contribution

[+] The membership list of POLAR_MI is provided in Additional File 1.

List of figures

Supplementary Figure 1. Retrieval request procedure resulting in the resource tables building the data snapshot 3

Supplementary Figure 2. Populations considered in POLAR_MI 4

Supplementary Figure 3. From a research question to the final analysis results 5

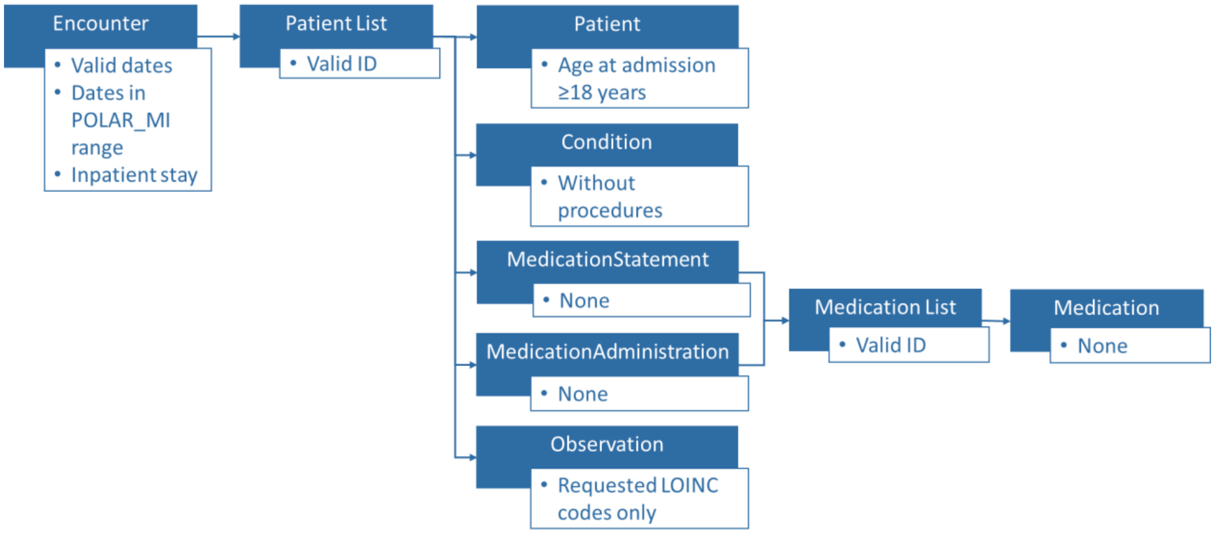

Supplementary Figure 1. Retrieval request procedure resulting in the resource tables building the data snapshot. FHIR resources (blue boxes) together with the required conditions for the entries (white boxes) are provided. For the overall workflow, see Figure 1. Abbreviations: FHIR, Fast Health Interoperability Resources; ID, identifier(s); LOINC, Logical Observation Identifiers Names and Codes.

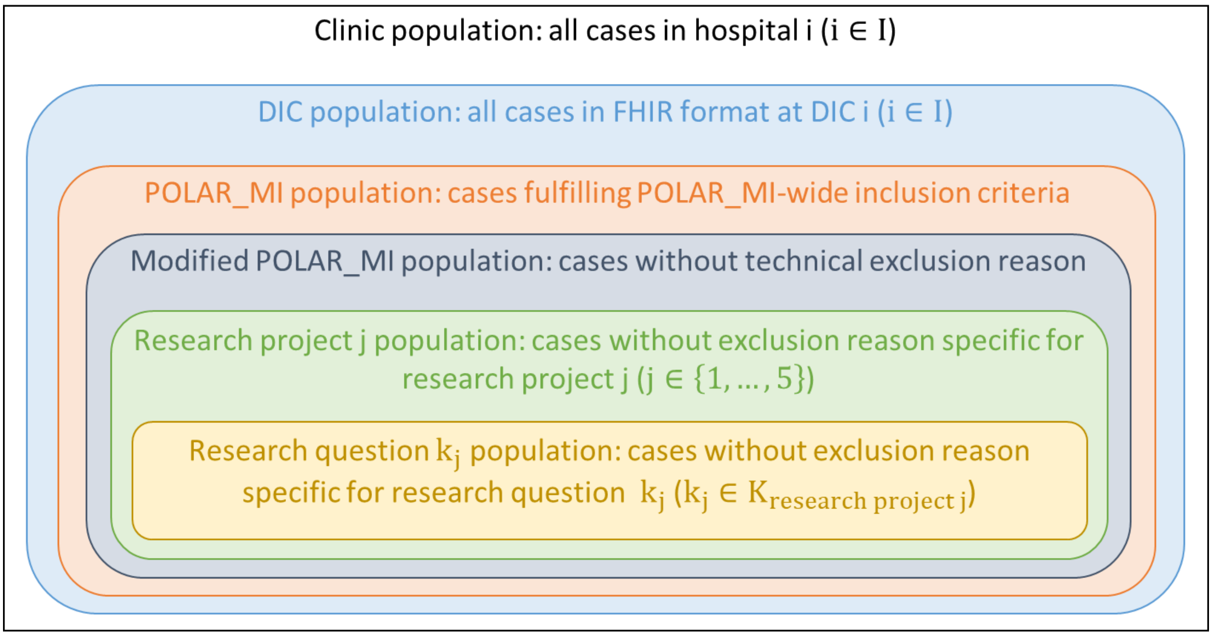

Supplementary Figure 2. Populations considered in POLAR_MI. Abbreviations: DIC, data integration centre; I, index set of participating centres; $K_{research\ project\ j}$, index set of defined research questions within the research project j ($j \in \{1, \ldots, 5\}$).

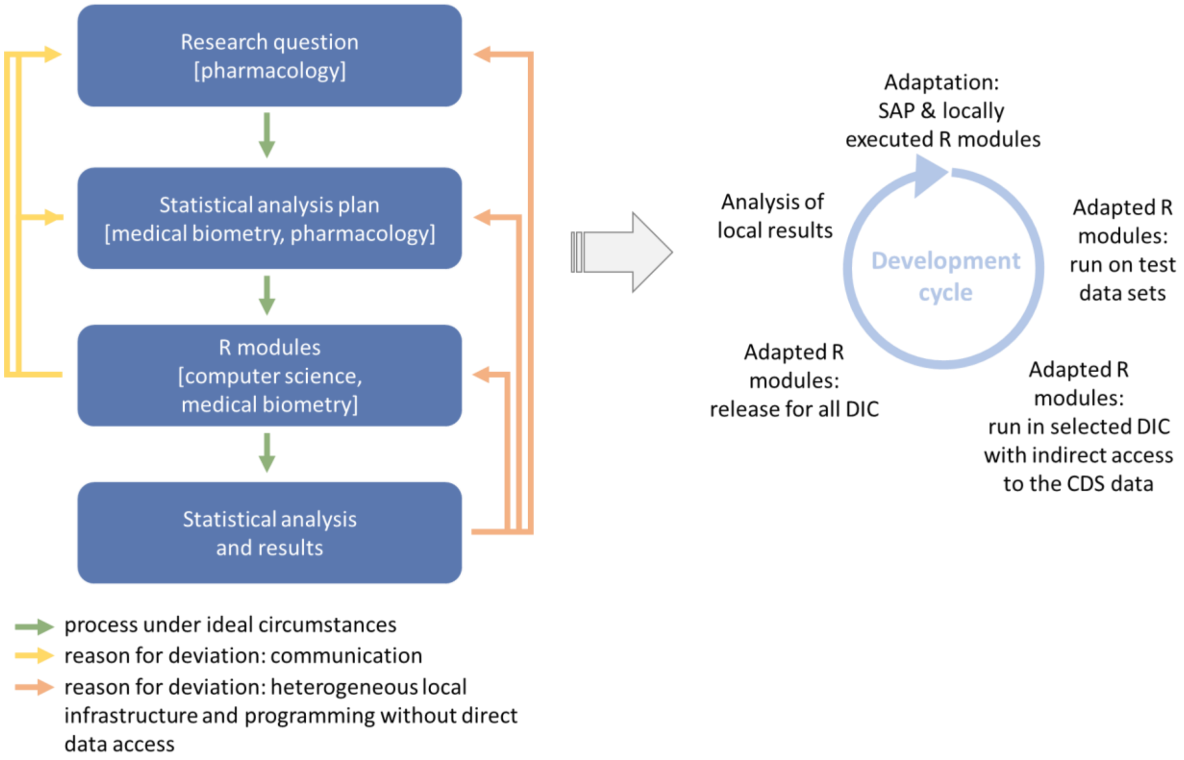

Supplementary Figure 3. From a research question to the final analysis results. Left-handed part: The process under ideal circumstances with required adaptations within POLAR_MI. Reasons for deviations from the straightforward development are given. Participating research disciplines are provided in squared brackets together with the respective task. Right-handed part: Introduced development/adaptation cycles of the statistical analysis plan and R modules. Abbreviations: CDS, core data set; DIC, data integration centre; SAP, statistical analysis plan.
