# Supplementary material for: A distributed analysis approach for pharmacovigilance data from electronic medical records in German university hospitals: the POLAR_MI ETL Pipeline
Source: BMC Med Inform Decis Mak. 2026 Jun 15;26:220. doi: 10.1186/s12911-026-03550-w (PMC13270667; doi:10.1186/s12911-026-03550-w)
Supplement: Supplementary file 4 — Supplementary Material 4: Additional File 4: Supplementary Methods (PDF) [file 12911_2026_3550_MOESM4_ESM.pdf]

Supplement S4: Supplementary Methods

A distributed analysis approach for pharmacovigilance data from electronic medical records in German university hospitals: the POLAR_MI ETL Pipeline

Miriam Kesselmeier[1,#], Torsten Thalheim[2,3,4], Florian Schmidt[3], Thomas Peschel[3], Julia Palm[1], Alexander Strübing[3], André Medek[5], Jens Przybilla[3,6], Anna Maria Wermund[7], Renke Maas[8], Steffen Härterich[9], Louisa Redeker[10], Martin Federbusch[11], Daniel Steinbach[11], Jan Gewehr[12], Marcus Wurlitzer[12], Andrea Riedel[13,14], Frank Meineke[3], Daniel Neumann[3], André Scherag[1,*] and Markus Loeffler[3,*] on behalf of POLAR_MI[+]

[1] Institute of Medical Statistics, Computer and Data Sciences (IMSID), Jena University Hospital – Friedrich Schiller University Jena, Jena, Germany
[2] Interdisciplinary Centre for Bioinformatics, Leipzig University, Leipzig, Germany
[3] Institute for Medical Informatics, Statistics and Epidemiology (IMISE), Leipzig University, Leipzig, Germany
[4] Deutsches Biomasseforschungszentrum gGmbH, Torgauer Str. 116, 04347 Leipzig, Germany
[5] Medical & Scientific Technology Development & Coordination (MWTek), University Hospital Bonn, Bonn, Germany
[6] Clinical Trial Centre Leipzig (ZKS), Leipzig University, Leipzig, Germany
[7] Department of Clinical Pharmacy, Institute of Pharmacy, University of Bonn, Bonn, Germany
[8] Institute of Experimental and Clinical Pharmacology and Toxicology, Friedrich-Alexander-Universität Erlangen-Nürnberg, Erlangen, Germany
[9] Hospital Pharmacy, University Medical Center Hamburg-Eppendorf, Hamburg, Germany
[10] Department of Clinical Pharmacology, School of Medicine, Faculty of Health, Witten/Herdecke University, Witten, Germany
[11] Institute for Laboratory Medicine, Clinical Chemistry and Molecular Diagnostics, University Medical Center Leipzig, Leipzig, Germany
[12] Business Division for Information Technology, University Medical Center Hamburg-Eppendorf, Hamburg, Germany
[13] Erlangen University Hospital, Medical Center for Information and Communication Technology, Erlangen, Germany
[14] Friedrich-Alexander-Universität Erlangen-Nürnberg, Medical Informatics, Erlangen, Germany

[#] Corresponding author
Email: miriam.kesselmeier@med.uni-jena.de

[*] Equal contribution

[+] The membership list of POLAR_MI is provided in Additional File 1.

List of content

Adapting/refining the approach for usage within POLAR_MI 3
  Overall workflow 3
  Description of the population available for the analyses 3
  Assumptions and definitions 3
References 4

Adapting/refining the approach for usage within POLAR_MI

Overall workflow

Within each research project, specific research questions were defined. Based on these research questions, the related statistical analysis was planned and specified in a statistical analysis plan (SAP). Subsequently, the modules for the local statistical analysis and the central meta-analysis were prepared and executed. Finally, the results were obtained. For an overview including the contributing research disciplines, we refer to Supplementary Figure 3 (Additional File 3).

Description of the population available for the analyses

For consistency and comparability reasons, we defined statistical descriptions for the POLAR_MI population, as well as for the modified POLAR_MI population and for each population of the five research projects (Supplementary Figure 2, Additional File 3). These POLAR_MI-wide descriptions comprised the following characteristics:

- Number of included encounters and patients
- Demographics: age and sex distribution
- Length of hospital stay (in the strict sense: time difference between start date and end date of an encounter)
- Medications: number of encounters with at least one medication and number of different medications per encounter (Supplementary Table 3, Additional File 2) (1)
- Diagnoses: number of encounters with diagnoses and number of different diagnoses per encounter (Supplementary Table 3, Additional File 2) (2, 3)
- Charlson comorbidity index (accounting for age) (4)

This summary was additionally stratified by age categories and sex. Furthermore, the following information was collected for descriptive purposes:

- Time interval covered by the encounters in the data snapshot
- Time point of data snapshot creation and local analysis execution
- Frequency of encounter exclusions together with their reasons (as a flowchart)

While these general, informative descriptions were consistently calculated for all POLAR_MI analyses, each research project complemented them with its project-specific descriptions to make the result interpretation feasible. For applications and examples, see (5, 6).

Assumptions and definitions

During implementation, we identified several difficulties related to the heterogeneity of local data (structure) and infrastructure at the DIC, to the CDS specifications and to professional, interdisciplinary exchange (Supplementary Figure 3, Additional File 3). Consequently, we needed to adapt our modules repeatedly. Furthermore, we had to make several assumptions in order to extract the required information for the analyses from the data. Missing information and the handling of non-evident default values used for the replacement of missing information were crucial. We had to deal with issues resulting from vague or redundant definitions, such as a missing specification of the SI units of laboratory values. Data transformation from HIS into FHIR contained assumptions itself. The details of our POLAR_MI-wide assumptions and definitions, which we required for the analyses, are presented as our results. Note that a specific research project within POLAR_MI might require additional assumptions and definitions, which are presented in the respective publications (5, 6).

References

1. Anatomisch-Therapeutisch-Chemischen Klassifikation (ATC-Klassifikation) mit definierten Tagesdosen (DDD) der Weltgesundheitsorganisation und den Anpassungen entsprechend der deutschen Versorgungssituation: Bundesinstitut für Medizinprodukte und Arzneimittel (BfArM) im Auftrag des Bundesministeriums für Gesundheit; [Accessed 2024/11/20]. Available from: https://www.bfarm.de/EN/Home/_node.html.
2. ICD-10-GM Version 2020, Systematisches Verzeichnis, Internationale statistische Klassifikation der Krankheiten und verwandter Gesundheitsprobleme, 10. Revision, Stand: 20. September 2019: Deutsches Institut für Medizinische Dokumentation und Information (DIMDI) im Auftrag des Bundesministeriums für Gesundheit (BMG) unter Beteiligung der Arbeitsgruppe ICD des Kuratoriums für Fragen der Klassifikation im Gesundheitswesen (KKG); 2019 [Accessed 2024/11/20]. Available from: https://www.bfarm.de/EN/Home/_node.html.
3. ICD-10-GM Version 2021, Systematisches Verzeichnis, Internationale statistische Klassifikation der Krankheiten und verwandter Gesundheitsprobleme, 10. Revision, Stand: 18. September 2020: Bundesinstitut für Arzneimittel und Medizinprodukte (BfArM) im Auftrag des Bundesministeriums für Gesundheit (BMG) unter Beteiligung der Arbeitsgruppe ICD des Kuratoriums für Fragen der Klassifikation im Gesundheitswesen; 2020 [Accessed 2024/11/20]. Available from: https://www.bfarm.de/EN/Home/_node.html.
4. Quan H, Li B, Couris CM, Fushimi K, Graham P, Hider P, et al. Updating and validating the Charlson comorbidity index and score for risk adjustment in hospital discharge abstracts using data from 6 countries. American Journal of Epidemiology. 2011;173(6):676-82.
5. Wermund AM, Thalheim T, Medek A, Schmidt F, Peschel T, Strübing A, et al. Challenges in detecting and predicting adverse drug events via distributed analysis of electronic health record data from German university hospitals. PLOS Digital Health. 2025;4(6):e0000892.
6. Redeker L, Kesselmeier M, Mussawy B, Grabe S, Rottenkolber M, Thalheim T, et al. Use of potentially inappropriate medication and association with falls during hospitalisation: an analysis based on electronic health records (POLAR_MI project). Drugs - Real World Outcomes. 2025.
