# Supplementary material for: A distributed analysis approach for pharmacovigilance data from electronic medical records in German university hospitals: the POLAR_MI ETL Pipeline
Source: BMC Med Inform Decis Mak. 2026 Jun 15;26:220. doi: 10.1186/s12911-026-03550-w (PMC13270667; doi:10.1186/s12911-026-03550-w)
Supplement: Supplementary file 5 — Supplementary Material 5: Additional File 5: Ethics committees and the reference numbers of the participating centres [file 12911_2026_3550_MOESM5_ESM.pdf]

Table 1. Ethics committees and the reference numbers of the participating centres.

Participating centre	Ethics Committee		Reference number
	Official German Name	Official English name	
Bonn	Ethikkommission an der Medizinischen Fakultät der Rheinischen Friedrich-Wilhelms-Universität Bonn	Ethics Committee at the Medical Faculty of the Rheinische Friedrich-Wilhelms-Universität Bonn	101/21
Erlangen	Ethik-Kommission an der Medizinischen Fakultät der Friedrich-Alexander-Universität Erlangen-Nürnberg	-	368_20 Bc
Freiburg/Breisgau	Ethik-Kommission der Albert-Ludwigs-Universität Freiburg	Ethics Committee of the Albert Ludwig University of Freiburg	20-1267
Gießen	Ethik-Kommission des Fachbereichs Medizin der Justus-Liebig-Universität Gießen	Ethics Committee of the Faculty of Medicine at Justus Liebig University Giessen	adopted the approval granted by the lead ethics committee (Leipzig)
Halle/Saale	Ethik-Kommission der Medizinischen Fakultät der Martin-Luther-Universität Halle-Wittenberg	Ethics Committee at the Medical Faculty of the Martin Luther University Halle-Wittenberg	2021-119
Hamburg	ETHIK-KOMMISSION DER ÄRZTEKAMMER HAMBURG	-	2020-10251-BO-bet
Heidelberg	Universität Heidelberg Ethikkommission der Medizinischen Fakultät	Ethics Committee of the Medical Faculty of Heidelberg University	S-240/2021
Jena	Universitätsklinikum Jena Ethik-Kommission	-	2020-1931-Daten
Kiel	Ethik-Kommission der Medizinischen Fakultät der Christian-Albrechts-Universität zu Kiel	Ethics Committee at the Faculty of Medicine of Kiel University	B 280/21
Leipzig (lead)	Ethik-Kommission an der Medizinischen Fakultät der Universität Leipzig	Ethics Committee at the Medical Faculty of the University Leipzig	247/20-ek
München	Ethikkommission der Ludwig-Maximilians Universität München	-	20-0961
Tübingen	Ethik-Kommission an der Medizinischen Fakultät der Eberhard-Karls-Universität und am Universitätsklinikum Tübingen	The Ethics Committee at the Medical Faculty of the Eberhard Karls University and at the University Hospital of Tübingen	712/2020BO2

Abbreviation: -, not available.
